# Supplementary material for: Dormancy Versus Germination: 3D Protein Modeling and Evolutionary Analyses Define the Roles of Genetic Variants in the Barley MKK3 Enzyme
Source: Int J Mol Sci. 2026 Jan 5;27(1):530. doi: 10.3390/ijms27010530 (PMC12787138; doi:10.3390/ijms27010530)
Supplement: Supplementary file 1 [file ijms-27-00530-s001.zip › ijms-4033614-Dormancy Versus Germination_Supplementary Data Set S1.pdf]

Hv_Morex.MAPK.kinaseDomain.aln.trimmed.fa

```
>HORVU.MOREX.r3.1HG0052130.1_kinase_48_330
---RPIGRGAYGIVCAAVSSDTGEEVAIKKIGNAFDNHIDAKRTLREIKLLRHMDHENIL
AMKDLIRPPRRDDFKDVYIVTELMDTDLHQIIRSNQSLTDDHCQYFLYQLLRGLKYVHSA
NVLHRDLKPSNLFLNANCDLKIADFGLARTTSETDLMTEYVVTRWYRAPELLLNCSQYTA
AIDVWSVGCILGEIITRQPLFPGRDYIQQLKLITELIGSPDDSSLGFLRSDNARRYMKQL
PQYPRQDFRLRFRNMSDGAVDLLERMLVFDPSRRITVDEALHHPYL
>HORVU.MOREX.r3.1HG0088960.1_kinase_34_325
YKIEIIGKGSYGVVCSAIDRQTGDKVAIKKISNIFEHITDAARILREIKLLRLLRHPDIV
QIKHIMLPPSRRDFKDIFVVFELMDTDLHQVIKANDDLTKEHFQFFLYQMLRAMKYIHTA
NVYHRDLKPKNILANANCKLKICDFGLARVTPTTVFWTDYVATRWYRAPELCGSFTKYSP
AIDTWSIGCIFAEILTGKPLFPGKNVVHQLDLMTDFLGSPSPDIISRIRNEKARRYLSTM
RKKLPVPFSEKFPNADPAAVKLLQKLLAFDPKDRPTAEEALADPYF
>HORVU.MOREX.r3.1HG0091240.1_kinase_133_425
YQVEVIGKGSYGVVAAAIDTQTGERVAIKKINDVFDHVSDATRILREIKLLRLLRHPDIV
QIKHIMLPPSRREFRDIYVVFELMESDLHQVIKANDDLTPEHHQFFLYQLLRGMKYIHAA
SVFHRDLKPKNILANADCKLKICDFGLARVAPSAIFWTDYVATRWYRAPELCGSFSKYTP
AIDIWSVGCIFAEMLTGKPLFPGKNVVHQLDLMTDVLGTPSAESLAKIRNEKARRYLSNM
RKKPKVPLTKKFPGIDPMALHLLERLLAFDPKDRPTADEALTDPYF
>HORVU.MOREX.r3.1HG0092210.1_kinase_168_459
YKIEVIGKGSYGVVCSALDLQTRQKVAIKKIHNIFEHTSDAARILREIKLLRLLRHPDVV
EIKHIMLPPSRKDFKDIYVVFELMESDLHQVIKANDDLTKEHYQFFLYQLLRALKYIHTA
SVYHRDLKPKNILANSNCKLKICDFGLARVTPTTVFWTDYVATRWYRAPELCGSFTKYTP
AIDIWSIGCIFAEVLTGKPLFPGKNVVHQLDLMTDLLGTPSMDTISRVRNEKARRYLTSM
RKKDPVPFSQKFPNADPLGVKLLEKLLAFDPKDRPTAEEALTDPYF
>HORVU.MOREX.r3.3HG0273800.1_kinase_25_316
YKIEIVGKGSYGVVCSAIDVHTGEKVAIKKIHDIFEHISDAARILREIKLLRLLRHPDIV
EIKHIMLPPSRRDFKDIYVVFELMESDLHQVIKANDDLTKEHYQFFLYQLLRALKYIHTA
NVYHRDLKPKNILANSNCKLKICDFGLARVTPTTIFWTDYVATRWYRAPELCGSFSKYTP
AIDVWSIGCIFAEVLTGKPLFPGKNVVHQLDLMTDLLGTPSMDTISRVRNEKARRYLTSM
RKKEPISFSHKFPNADPLALDLLQRLLAFDPKDRPTAEEALSHPYF
>HORVU.MOREX.r3.3HG0275260.1_kinase_21_312
YEVEVVGKGSYGVVAAAVDTHTGERVAIKKIDDVFEHVSDATRILREVKLLRLLRHPDIV
QIKHIMLPPSRREFRDIYIVFELMESDLHQVIKANDDLSPEHHQFFFYQLLRGMKYIHAA
NVFHRDLKPKNILANADCKLKICDFGLARVTPSAIFWTDYVATRWYRAPELCGSFSKYTP
AIDIWSIGCIFAEMLSGRPLFPGKNVVHQLDLMTDLLGTPSAESISRIRNEKARRYLGNM
KKKHPIPFSQKFPGVDPMALHLLERLLAFDPADRPTAAEALADPYF
>HORVU.MOREX.r3.3HG0278110.1_kinase_30_321
YKIEVIGKGSYGVVCSAIDQHNGDKVAIKKIHNIFEHLSDAARILREIKLLRLLRHPDIV
EIRHIMLPPSRRDFKDIYVVFELMDTDLHQVIKANDDLTKEHHQFFLYQMLRALKYIHTA
NVYHRDLKPKNILANANCKLKICDFGLARVTPTTVFWTDYVATRWYRAPELCGSFTKYSP
AIDIWSIGCIFAEILTGKPLFPGKNVVHQLDLMTDLLGTPSLDTVSRIRNEKARRYLSSM
RKKQTVCFSERFPKADPAALKLMQRLLAFDPKDRPTAEEALADPYF
>HORVU.MOREX.r3.4HG0385780.1_kinase_41_322
----PIGRGAYGIVCSVMNFETREMVAIKKIANAFDNNMDAKRTLREIKLLKHLDHENIV
GLRDVIPPAIPQSFNDVYIATELMDTDLHHIIRSNQELSEEHCQYFLYQLLRGLKYIHSA
NVIHRDLKPSNLLLNANCDLKICDFGLARPSSESDMMTEYVVTRWYRAPELLLNSTDYSA
AIDVWSVGCIFMELINRAPLFPGRDHMHQMRLITEVIGTPTDDDLGFIRNEDARRYMRHL
PQFPRRPFPGQFPKVQPAALDLIERMLTFNPLQRITVEEALEHPYL
```

```
>HORVU.MOREX.r3.5HG0495500.1_kinase_1_130
------------------------------------------------------------
------------------------------------------------------------
------------------------------------MTEYVVTRWYRAPELLLSFDKYGT
SVDVWSVGCIFAELLGRKSIFPGTDCLNQLKLIVDVLGTMSDANLEFIGNTKARKYVKSL
SYTVGVPLTRMYPQAHPLAIDLLEKMLVFDPSKRISVTEALEHPYM
>HORVU.MOREX.r3.5HG0533360.1_kinase_4_283
YKVREIGDGTCGNVFRAYNIETNEIVAVKKMKRKFYHWEECI-SLREVKALQKLNHPNIV
QLKEVT-----MENHELFFIFEHMDCNLYDVIRERAPFSEEEIRKFMLQILQGLVYMHNN
GYFHRDLKPENLLVSNGI-VKIADFGLAREVCSTPPYTDYVSTRWYRAPEVLLQASAYTP
SIDMWAIGAILAELFTLSPLFPGETETDQLFKICAVLGTPDHSLWPEGMN-LPRSSSFQF
FQIPPRNLWELIPNASLEALDLIKQLCSWDPRRRPTAEQALQHPFF
>HORVU.MOREX.r3.6HG0555110.1_kinase_13_304
YQIEIIGKGSFGVVAAAVDTQTGEWVAIKKIHDMFEHASDGTRILREIKLLRLLRHPNIV
EIKHILLPPARREFRDIYVVFELMESDLQKVIQVNDNLTKGHHRFFLYQLLHALKYIHTA
NVFHRDLKPSNILANSNCKLKICDFGLARAVPSAIFWTDYVATRWYRAPELCGSFSKYTP
AIDIWSIGCIFAEVLTRNPLFPGTNVTHQLDLITDVLGTPSHETLSQIRNEKARRYLTGM
KRKHPIPFPRMFCSADPQAVRLLERLLAFDPKDRPTAEEALADPYF
>HORVU.MOREX.r3.6HG0559130.1_kinase_33_319
VPIKPIGRGAYGIVCSSINRETNEKVAIKKIHNVFDNRVDALRTLRELKLLRHLRHENVI
SLKDIMMPVQRRSFKDVYLVYELMDTDLHQIIKSPQGLSNDHCQYFLFQLLRGLKYLHSA
EILHRDLKPGNLLVNANCDLKICDFGLARTSSKGQFMTEYVVTRWYRAPELLLCCDNYGT
SIDVWSVGCIFAELLGRKPIFPGTECLNQLKLIVNVLGTMSESDLEFIDNPKARRYIKTL
PYTPGVPLASMYPHAHPLAIDLLQKMLIFDPTKRISVTQALEHPYM
>HORVU.MOREX.r3.6HG0606710.1_kinase_5_283
-KLKEVGDGTFGSVWRAINKQNGEVVAVKKMKRKYYSFEECM-SLREVKSLRRMNHPNIV
KLKEVI-----RENDILYFIMEYMECNLYQLMKDRKPFAESDVRNWCFQIFQALAYMHQR
GYFHRDLKPENLLVSKDV-LKLADFGLAREVSSAPPYTEYVSTRWYRAPEVLLQSSAYDS
AVDMWAMGAIMAELLTLHPLFPGTSEADEILKICNVIGSPDEQTWPQGLS-LAEAMKYQF
PQIRGNQLSEVMKSASSEAVDLISSLCSWDPCKRPKAAEVLQHAFF
>HORVU.MOREX.r3.7HG0641060.1_kinase_4_283
YNITEVGDGTFGSVWRAINKESGEVVAIKKMKKKYFSWEECI-NLREVKSLRRMNHPNIV
KLKEVI-----RENDMLFFVFEYMECNLYQLMKSKKPFSETEIRNWCFQVFQALSHMHQR
GYFHRDLKPENLLVTKEL-IKVADFGLAREIISEPPYTEYVSTRWYRAPEVLLQSSVYSS
AVDMWAMGAIIAELFSHRPLFPGSSEADEIYKICNILGTPNQHTWAGGLQ-LAASIHFQF
PQSGSINLSEVVPTASEDALNLISWLCSWDPRKRPTAVEVLQHPFF
>HORVU.MOREX.r3.7HG0652970.1_kinase_69_348
-----IGKGAYGIVCSALNSETGEQVAIKKIANAFDNKIDAKRTLREIKLLRHMDHENIV
AIRDIIPPAQRTAFNDVYIAYELMDTDLHQIIRSNQALSEEHCQYFLYQILRGLKYIHSA
NVLHRDLKPSNLLLNANCDLKICDFGLARTTSETDFMTEYVVTRWYRAPELLLNSSEYTA
AIDVWSVGCIFMELMDRKPLFPGRDHVHQLRLLMELIGTPNEADLDFV-NENARRYIRQL
PRHARQSLSEKFPHVHPSAIDLVEKMLTFDPRQRITVEGALAHPYL
>HORVU.MOREX.r3.7HG0712330.1_kinase_26_317
YKVEVIGKGSYGLVCSANDTQTGEKVAIKKIHNIFEHISDAARILREIKLLRLLRHPDVV
EIKHILLPPSKKDFKDIYVVFELMESDLHQVIKANDDLTREHYQFFLYQMLRALKYMHTA
NVYHRDLKPKNVLANANCKLKICDFGLARVAPTTVFWTDYVATRWYRAPELCGSYSKYTP
AIDIWSIGCIFAEVLIGKPLFPGKNVVHQLDLITDVLGTPSLDAISQVRNDKARKYLTYM
RKKQPASFSQKFPKADPLALRLLRRLLAFDPKDRPSAEEALADPYF
>HORVU.MOREX.r3.7HG0726170.1_kinase_87_378
YQIEVVGKGSYGVVAAAIDTRTGERVAIKKINDVFEHVSDATRILREVKLLRLLRHPDVV
```

```
EIKHIMLPPSRREFQDIYVVFELMESDLHQVIRANDDLTAEHYQFFLYQLLRALKYIHGA
NVFHRDLKPKNILANADCKLKICDFGLARVAPSAIFWTDYVATRWYRAPELCGSFSKYTP
AIDIWSIGCIFAELLTGRPLFPGKNVVHQLDIITDLLGTPSSETLSRIRNEKARRYLSCM
RKKHPVPLTQKFPNADPLAVRLLGRLLAFDPKDRPSAEEALADPYF
>HORVU.MOREX.r3.7HG0728160.1_kinase_33_319
VPIKPIGRGAYGIVCSSINQETNEKVAIKKINNVFDNRVDALRTLRELKLLRHLRHENVI
ALKDIMMPIHRRSFKDVYLVSELMDTDLHQIVKSSQPLSNDHCQYFLFQLLRGLKYLHSA
GILHRDLKPGNLLVNANCDLKICDFGLARTNTKGQFMTEYVVTRWYRAPELLLCCDNYGT
SIDVWSVGCIFAELLGRKPIFPGTECLNQLKLIVNVLGTMSEADLAFIDNSKARKYIKSL
PYTPGIPLSSMYPQAHPLAIDLLQKMLVFDPSKRISVTQALEHPYM
```

Hv_Morex.MAPKK.kinaseDomain.aln.trimmed.fa

```
>HORVU.MOREX.r3.1HG0086640
-ADLGEGASGVVTKVRLRGTAAGPALALKVAHYPDD-TDAGREQEVLRRACSPYVVRCHA
VLRAAGEPACLLELMDAGSLHDVRALPEPAVAEVAARCALGLAHLHARGVAHLDLKPDNL
LAGARGDVKIADFGVSRIFQRRPPRVSIAVGTTAYMSPERFAPNAQAGSRGACAADVWSL
GVTVLELFLGHRPVLP-AERTPSWKMLKEAICYGEPPSVPAAASAELRGFVAACVQKDPR
RRATVPQLLAHPFV
>HORVU.MOREX.r3.4HG0395160
ISQLGQGNGGTVFRARHRRTSQHFALKLFAAGDGDP--SAAREAEILMLASAPHVVRLHA
VIPAAAQPALALELVSGGSLAGLRPMGERPIAAVARQALLGLEALHALRVVHRDLKPANL
LVGPGGEVKIADFGAGKVLRRRLDPCASYVGTAAYMSPERFDPETYAGDYDPYAADVWSL
GMAILELYLGHFPLLP-AGQRPDWAALMCAICFGEAPEAPAAASDEFRDFVARCLEKKAG
RRASVAELLEHPFI
>HORVU.MOREX.r3.5HG0485450
--------------VRHRPTTRFYALKVLYGNHDDARREIARDIAILRTAEHPVVVRCHD
MCERGGELQILLEYMDGGSRDGCRIAYEPFLAHVARQVLSGIAYVHRRHIVHCDIKPSNL
LIDSARRVKIADFRVGRILNKTMDPCNSSVGTIAYMSPERINTDINDATYDGYAGDIWSF
GLSILEFYLGRFPFGENLGRQGDSA-----------------------------------
--------------
>HORVU.MOREX.r3.5HG0537390
FGPIGNGASSVVQRAIFIPVHRILALKKINIFEKEKRQQILNEMRTLCEASYPGLVEFQG
AFYDSGQISIALEYMDGGSLADVKSIPEPVLAHMLLKVLLGLKYLHERHLVHRDLKPANI
LVNLKGEAKITDFGVSAGLDNTMAMCATFVGTVTYMSPERIRNENY-----SYAADIWSL
GLTILECATGKFPYNVNEGP----ANLMLQILDDPSPAPPDAYTPEFCSFINDCLRKDAD
ARPTCEQLLSHPFI
>HORVU.MOREX.r3.5HG0537440
FGPIGNGASSVVQRAIFIPVHRILALKKINIFEKEKRQQILNEMRTLCEASYPGLVEFQG
AFYDSGQISIALEYMDGGSLADVKSIPEPVLAHMLLKVLLGLKYLHERHLVHRDLKPANI
LVNLKGEAKITDFGVSAGLDNTMAMCATFVGTVTYMSPERIRNENY-----SYAADIWSL
GLTILECATGKFPYNVNEGP----ANLMLQILDDPSPAPPDAYTPEFCSFINDCLRKDAD
ARPTCEQLLSHPFI
>HORVU.MOREX.r3.6HG0622200
VRRVGSGAGGTVWLVRHAPTGRAYALKVLYGHHDEARRQITREIAILRTAEHPSIVRCHG
MYEQAGELQILLEYMDGGSLDGRRIASEVFLADVARQVLSGIAYLHRRHIVHRDIKPSNL
LIDSGRRVKIADFGVGRILNQTMDPCNSSVGTIAYMSPERINTDLNDGNYNGYAGDIWSF
GLSILEFYLGRFPLGENLGKQGDWAALMCAICYSESPAAPPTASPELRSFISCCLQKNPA
KRPSAAQLLQHRFI
>HORVU.MOREX.r3.7HG0660310
VRRVGSGAGGTVWMVRHRPTARCYALKVLYGNHDDARRQIAREIAILRTAEHPAVVRCHG
MYERGGELQILLEYMDGGSLDGRRIADEPFLAHVARQVLSGIAYLHRRHIVHRDIKPSNL
LIDSARRVKIADFGVGRILNQTMDPCNSSVGTIAYMSPERINTDINDGAYDGYAGDIWSF
GLSILEFYLGRFPFGENLGRQGDWAALMVAICYSDPPPSPATASPEFRGFIACCLQKNPA
KRLSAAQLLQHPFV
```

Hv_Morex.MAPKKK.kinaseDomain.aln.trimmed.fa

```
>HORVU.MOREX.r3.1HG0000080.1_kinase_24_225
SNLLGEGGFGSVYKGRLEGKDVAVKQLRDGSQGEREFQAEVEIISRVHHRHLVSLVGYCI
ANSQRLLVYDFVSNDTLHYHLHGRVLEWPARVKIAAGAARGIAYLHEDPRIIHRDIKSSN
ILLDNNFDALVADFGLARLATTRVMGTFGYMAPEYASSGKLTEKSDVFSFGVVLLELMTG
RKPVD--------------------------------
>HORVU.MOREX.r3.1HG0001280.1_kinase_15_197
------------------------------------------------------------
----------------------RRLLDWGARQNIIHGTARGLLYLHEDIKILHRDLKASN
ILLDASMIPKISDFGLARLFTSQVVGTLGYMAPEYAVLGHLSVKLDVYSFGVLVLEIVSG
RRSTDHVMDPTLDGETCIHIALLCVQANPANRPTMLD
>HORVU.MOREX.r3.1HG0004150.1_kinase_28_283
TEVLGKGAFKTVYKAFDEGLEVAWNQIKVGDRNLERLRSEVRLLKTLKHKNIIKFYNSWD
KKNNNIFITEVFTSGTLRQYRIKK-VDIRALKKWSRQILSGLVYLHGHPPIIHRDLKCDN
IFVNGN-EVKIGDLGLATI-LDSIIGTPEFMAPELYDEE-YNELVDIYAFGMCLLELVTF
EYPYCNVSDGEKPGSLVKFFIEKCIAQ-ASQRLSAEE
>HORVU.MOREX.r3.1HG0006430.1_kinase_530_792
NKKLGEGGFGSVYEGKL-GERVAVKRLESARQGKKEFLAEVQTIGSIEHINLVRLIGFCA
EKSQRLLVYEYMPRGSLDRWIHNNPLDWPTRCRIILDIAKGLCYLHEEQKIAHLDIKPQN
ILLDEKFNAKVADFGLSKLIMTVMRGTPGYMAPEW-LTSQITEKVDVYSFGVVVMELISG
RKNIDELIDKHSEDMVKMKLAMWCLLNDGCQRPSMST
>HORVU.MOREX.r3.1HG0012180.1_kinase_45_313
--LIGRGAFSVVYMGVLSGLQIAVKKL-YPLGSEIAFENEVLTTMRVAHKNVVRLVGYCS
HTQQRLICTEYVPHGTLDRHII--VLDWNHRYKVLKGICHGLRYLHDE-RIVHKDIKESN
ILLGDSLVPKIFDFGFSTSLDNHIMGTIGYMAPELIIDRTYSFKSDIFSFGVVMMKLLIG
SRENH-RLVKGAF-AVCLQIVYSCMDMDPNNRPTALE
>HORVU.MOREX.r3.1HG0016450.1_kinase_344_571
INKLGQGGYGSVYKGVLDGREIAVKRLYLNTQWTDQFFNEVKLVSQVQHKNLVKLLGCSV
EGPESLLVYEYLCNTSLDHYLFKKALDWERRSEIVLGAAEGLSYLHSGVRIIHRDIKASN
VMLDERFRPKIGDFGLARNFSTGLAGTFGYMAPEYIVHGQLTEKADIYSYGVLVLEIVTG
RKNHNS-------------------------------
>HORVU.MOREX.r3.1HG0027350.1_kinase_2_136
------------------------------------------------------------
---------------------------------------------------VHGDIKGRN
VVVGADG-AKLADFGCARA-AAG--GTPTFMAPEVARGEEQGPPADVWALGCTVLEMATG
RIPWADIGFTDAVPEVAKDFLERCLRRRASDRATVAQ
>HORVU.MOREX.r3.1HG0028340.1_kinase_4_122
------------------------------------------------------------
------------------------------------------------------------
----ADG-AKLADFGCARA-AGA--GTPAFMAPEVARGEEQGRAADVWALGCTVIEMATG
RAPWG-IGYTDAVPELAKDFLRKCLARNPRHRPTAAE
>HORVU.MOREX.r3.1HG0037210.1_kinase_680_927
DERIGIGSYGEVYHADWNGTEVAVKKFLDQE--LEEFRCEVRIMRRLRHPNIVLFMGAVT
------IVSEYLPRGSLYKIIHQ--IDEKRRIKMALDVARGMNCLHTSPTIVHRDLKSPN
LLVDDNWTVKVCDFGLSRLKSSSTAGTPEWMAPEVL-RNQSNEKCDIYSFGVILWELATL
RKP--GVGFQDRRLDIVASIIRDCWQKDPNLRPSFIQ
>HORVU.MOREX.r3.1HG0050630.1_kinase_26_287
MEEVGYGAHAVVYRALFRNHTVAVKCLDLD-QLIDEVQREAQIMSLIDHPNVIRAYC--F
VVEHSLVIMPFMTEGSCLHLMKAYGFEEPVIASILKETLKALEYLHR-QGQIHRDIKAGN
```

ILIDSAG-VKLGDFGVSACM-DTFVGTPCWMAPEVLQPGGYNFKADIWSFGITALELAHG
HAPFSP-TLQNAPPGLFKEMVAMCLVKDQTKRPTAEK
>HORVU.MOREX.r3.1HG0067630.1_kinase_525_727
DNKLGEGGYGPVYKGKLDGQEIAVKTLSQASQGPDEFKNEVMLIAKLQHRNLVRLIGCCI
CGQEKILIYEYMENKSLDFFLFRSLLDWQTRYRIIEGIARGLLYLHQDYRIVHRDLKTSN
ILLDKDMTPKISDFGMARIFTLRVVGTYGYMAPEYAMDGVFSVKSDVFSFGVIVLEIITG
IRNRG--------------------------------
>HORVU.MOREX.r3.1HG0072410.1_kinase_73_331
DRELGRGEFGVTYLCMDSKELLACKSISKR-RTVEDVRREVAIMRHLRSASIVTLRE--C
EDDGAVLVMELCEGGELFDRIVARHYTERAAAAVTRTIVEVVQLCHH-HGVIHRDLKPEN
FLFANK-PLKAIDFGLSIFF-PEIVGSPYYMAPEVLKRN-YGPEIDIWSAGVILYILLCG
VPPFWQIDFKREPPH-AKDLVRQMLQPDPKIRLTAKQ
>HORVU.MOREX.r3.1HG0075260.1_kinase_366_565
ENKLGEGGFGPVYKGTLDGQEIAVKRLSKTSQGLVEMRNEVVLVAKLQHKNLVRLLGCCI
QEEEMLLVYEFLPNRSLDKILFRRELTWGHRFRIIQGIGRGLLYLHEDLTIIHRDLKASN
ILLDPDMNPKISDFGLAKLFTSHIAGTYGYMAPEYALHGIFSAKSDVYSYGVLVLEIVAG
RR-----------------------------------
>HORVU.MOREX.r3.1HG0076630.1_kinase_696_1001
ENLIGKGGSGRVYRVEC-STVVAVKRIWTGGEREREFESEVDVLGHVRHTNIVKLLCCLS
RAETKLLVYEYMDNGSLDKWLH-RPLDWPARVRVAVGAARGLSYMHHEPPVVHRDVKCSN
ILLDSELNAKVADFGLARILMSAVAGTFGYMAPECAYTRKANEKVDVYSFGVVLLELATG
REAGSHAADECIGDAFVFKLGIICTGAQPSTRPTMKD
>HORVU.MOREX.r3.1HG0077130.1_kinase_297_543
-----SGAYSRLFHGIYKEQPVAVKFIRQPDAAEKQFTVEVTILARLQHRNVIELVGACR
------VITEFLPGGSLRSFLRKQ-LPLDKILSIALDIARGLEYVHSQ--VIHRDVKPEN
ILFDAECCAKVVDFGVAFENLE-DPGTYRWMAPEMC-KRPYGPKVDVYSFGLVLWELVSG
SIP--EVVNKNLRPIVLQQLMEQCWSAQPEKRPVFTE
>HORVU.MOREX.r3.1HG0077360.1_kinase_758_972
TKLLGQGGFGHVYRGTLAGREVAIKRLRVDSQGDREFRAEVESIGRVHHRNLVSLVGYCI
HGDQRLLVYEHVSNHTLESHLHEPLLDWERRWRIALGAAKGLAYLHEDPKIIHRDIKAAN
ILLDDNFEPKVADFGLAKIQSTRVMGTFGYMAPEYTNTGKITDRSDVFSFGVVLLEIITG
KRPVLD-------------------------------
>HORVU.MOREX.r3.1HG0079430.1_kinase_11_260
VRTLGRGASGAVFLAADSGELFAVKS-----AAAAALRKERCVMATLSSPRIVSCIGGRG
AGDGSYLFLEFAPGGSLAEQVASN-LDERSVRGYALDVAAGLAYLHA-AGMVHGDVKSRN
VVIGADG-AKLADFGCSRK-SGG--GTPAFMAPEVARGEEQGPAADVWALGCMVVEMATG
HAPWSGIGYTEAMPEVGKDFLASCLVRQASGRCTAAQ
>HORVU.MOREX.r3.1HG0079460.1_kinase_50_298
VRTLGRGASGAVFLAADSGELFAVKS-----TAAAALRREQRVMAGLRSPRVVSCIGGRG
DRDGSYLFLEFAPGGSLADRVASN-LDELAIRGYAADIASGLAYLHS-AGMVHGDVKARN
VVIGADG-AKLADFGCARE-AAG--GTPAFMAPEVARGEEQGPAADVWALGCTVVEMATG
RAPWTGIGYTEAVPEVAKDFLRGCLVRQAGKRCTAAQ
>HORVU.MOREX.r3.1HG0079470.1_kinase_11_262
VRTLGRGASGAVFLAADSGELFAVKS-----APAAALRRERCVMATLSSPRIVSCIGGRG
AGDGSYLFLEFAPGGSLAEQVASN-LDERSVRGYALDVASGLTYLHA-AGMVHGDVKSRN
VVIGADG-AKLADFGCSRK-AAG--GTPAFMAPEVARGEEQGPAADVWALGCMVVEMATG
RAPWSGIGYTEAVPEVGKDFLASCLVRQASGRCTAVQ
>HORVU.MOREX.r3.1HG0079500.1_kinase_55_306
VRTLGRGASGAVFLAADSGELFAVKS-----APAAALRRERCVMATLSSPRIVSCIGGRG
AGDGSYLFLEFAPGGSLAEQVASN-LDERSVRGYALDVASGLTYLHA-AGMVHGDVKSRN

VVIGADG-AKLADFGCSRK-AAG--GTPAFMAPEVARGEEQGPAADVWALGCMVVEMATG
RAPWSGIGYTEAVPEVGKDFLASCLVRQASGRCTAAQ
>HORVU.MOREX.r3.1HG0079540.1_kinase_8_205
VRTLGRGASGAVFLAADSGELFAVKS-----AAAAALRREQCVMATLSSPRVVSCIGGRG
AGDGSYLFLEFAPGGSLADRMASK-LDERSLRGYALDVAAGLAYLHA-AGMVHGDVKSRN
VVIGADG-AKLTDFGCARD-AAG--GTPAFMAPEVARGEEQGPAADVWALGCTVVEMATG
RAPWSG-------------------------------
>HORVU.MOREX.r3.1HG0079600.1_kinase_17_265
VRTLGRGASGAVSLFAAGDELLAVKS-----AAAAQLRREGGILASLCSPYVLPCLGSRA
AAGGEYLFLEFAPGGSLADEVERNCLEEGAVRAYAADVARGLAYLHG-ESMVHGDVKGRN
VVIGADG-AKLADFGCARS-VGG--GTPAFMAPEVARGEEQGPAADVWALGCTVIEMATG
RAPWS-IGYTDAVPEAAKDFLDKCFRRCAGERWTAEQ
>HORVU.MOREX.r3.1HG0081230.1_kinase_518_789
NEELGRGGSGVVYRGVLKTTVVAVKELTNVVQGEEEFWAEMTVFGRINHINLVRIWGFCC
EGKHKLLVYEYVENGSLDKHLFISALTWSERFKVALGAARGLAYLHHEEWVIHCDVKPEN
ILLTRELDPKIADFGLSKLSLSHMRGTTGYMAPEWVLGLPVDAKVDVYSYGIVLLEILMG
SRITEDLVDSRLNGQAMVKISLSCM-EERSKRPTMDD
>HORVU.MOREX.r3.1HG0081250.1_kinase_513_780
NEELGRGGSGVVYRGVLKTTVVAVKELTNVVQGDEEFWAEMTVFGRINHINLVRIWGFCS
EGKHKLLVYEYVENLSLDRHLFNGALAWRERFKIALGTARGLAYLHHEEWVIHCDVKPEN
ILLTRDLDPKIADFGLSKLSLSHMRGTTGYMAPEWVLGLPIDAKVDVYSYGVVLLEILMG
SRITEDLVDSRLNGQAMLKISLSCM-EERSNRPAMD-
>HORVU.MOREX.r3.1HG0087290.1_kinase_380_653
-DVLGRGAYCTVYRGHLDGRAVAVKQL----YGEVEFWSEVTIMARMNHRNIVSIWGWCA
DKGQRMLILEFISNGSLDKHVLQRHLDLNTRHQIALGVAHAMAYMHEEDGVLHCDIKPHN
ILLDDNFCPKLTDFGLSTWLMSRVRGTRGYMAPEWVIHRVITAKADVYSFGMVLLEVVSG
-RNYNDILDPILQ--IMVNTAIWCLQDRAEKRPSMGK
>HORVU.MOREX.r3.1HG0089010.1_kinase_378_586
ENKLGEGGFGPVYKGILDGQEMAVKRLSKTSQGLVEMRNEVVLVAKLQHKNLVRLLGCCI
QEQEMLLVYEFLPNRSLDKILFRRELTWGHRFRIIQGIGRGLLYLHEDLTIIHRDLKASN
ILLDPDMNPKISDFGLAKLFTSHIAGTYGYMAPEYALHGIFSAKSDVYSYGVLVLEIIAG
RRNNF---------------------QHPGT------
>HORVU.MOREX.r3.1HG0091430.1_kinase_65_331
--VIARGTFGTVHRGVYDGLDVAVKLLDWGEITRAAFSQEVSVWHKLDHPNVTKFIGAIM
GADDGNVIVEYLAGGALKTFLIRK-LAFKVVVQIALDLARGLSYLHSK--IVHRDVKTEN
MLLDKTRTVKIADFGVARHEMT-ETGTLGYMAPEVL-NGAYNRKCDVYSFGICLWEVYCC
DMP--DVVRQNLRPEIFANVMKRCWDANPDKRPEMAE
>HORVU.MOREX.r3.1HG0092560.1_kinase_376_583
SNKLGDGGFGAVYKGILDGDEIAVKRLSKSSQGVEELKNELSLVAKLRHKNLVTLLGVCL
EQQERLLVYEFVPNRSLDLFLFKHELDWEMRYKIINGIARGLQYLHEDLRVVHRDLKASN
ILLDKDMNPKISDFGIARIFTNRVVGTYGYMAPEYVMRGNYSVKSDSFSFGVMVLEIVTG
RKNNE-----YSNSE---------------------
>HORVU.MOREX.r3.1HG0092660.1_kinase_358_568
SNKLGEGGFGPVYKGTLSGDEIAVKRMSKTSQGVEELKNELALVAKLKHKNLVRLVGVCL
EQQERLLIYEFVPNRSLDLILFKRQLDWETRFKIINGVARGIQYLHEDLKVVHRDLKASN
VLLDMNMNPKISDFGLAKIFTNRVIGTYGYMAPEYVMRGNYSVKSDAFSFGVLVLEVVTG
KKNSD-----CCNTQ---------------------
>HORVU.MOREX.r3.1HG0092670.1_kinase_348_611
ANKLGEGGYGIVYKGILDGQEIAVKKLLGATHGLHQLRNEVLLLAELQHKNLVRLQGFCS
HRDDTLLVYEYIKNGSLDNFLFEETLNWEQRYNIILGIAKGILYLHEDPRIIHRDLKANN

ILLDEEMDPKIADFGLARLLTTRAAGTLGYMAPEYAVHGSVSPKIDIFSFGVLVLEIITR
RRNCSDMMDESLNGYACIHIGLLCVQSDPDDRPQIST
>HORVU.MOREX.r3.2HG0099700.1_kinase_62_264
-CKIGHGGFGKVYKGMLGGQEVAIKRLSKDSQGTKEFSNEVILIAKLQHRNLVQLLGCCA
DGDEKLLIYEYMPNKSLDATLFRKLLDWSTRFNIIKGIARGILYLHQDLTIIHRDLKAGN
ILLDAEMKPKIADFGMARIFTQRVVGTYGYMAPEYAMEGLFSTKSDVYSFGVLLLEVVTG
IRRNS--------------------------------
>HORVU.MOREX.r3.2HG0102630.1_kinase_64_326
ENEIGRGGFGIVYKGQMDGQEIAVKKLSTNNQGMKEFKNEVDLIARLQHRNLVRLLGCCI
HYSDRILVYEYMSNKSLDNFIFRRTLSWKTRMDIILGVARGLLYLHQDHTMIHRDLKAAN
VLLNGEMVAKISDFGIAKLFTERIVGTFGYMAPEYAMDGMVSFMQDVYSFGVLLLEIVSG
RRNQR-LLDPTVRDDLCIQVGLLCVQESPSQRPQM--
>HORVU.MOREX.r3.2HG0102650.1_kinase_470_734
ENEIGRGGFGIVYKGQMDGQKIAVKKLSTNNQGMKEFKNDVDLIARLQHRNLVRLLGCCI
HYSDRILVYEYMSNKSLDNFIFRRTLSWKTRMDIILGVARGLLYLHQDHTMIHRDLKAAN
VLLNGEMVAKISDFGIAKLFTERIVGTFGYMAPEYAMDGMVSFMQDVYSFGVLLLEIVSG
RRNQR-LLDPTVRDDLCIQVGLLCVQESPSQRPQM--
>HORVU.MOREX.r3.2HG0104600.1_kinase_3_134

-------------NGSLERWLHPNTLSLRLRICIAMDVASALDYLHSEPALMHCDLKPSN
ILLDYDMTARIGDFGSAKF-AENFRGTIGYMAPEYGMGCQISAGGDVYSFGVILLELLTG
KRPT-D-------------------------------
>HORVU.MOREX.r3.2HG0109850.1_kinase_6_256
VELVGEGSFGKVYKGRRSRQTVAMKFILKH-SDHN-LRQEIEILRKLKHENIIEMID--F
ETPQEFVVTEFAQ-GELFEVLEDDCLPEEQVQAIAKQLVKALHYLHS-NRIIHRDMKPQN
ILI----IVKLCDFGFARAM-ASIKGTPLYMAPELVREQ-YNHTADLWSLGVILYELFVG
QPPFYYVKY----PDNFKSFLKGLLNKLPQSRL----
>HORVU.MOREX.r3.2HG0111160.1_kinase_3_68

--------PKIADFGMARIFTQRVVGTYGYMAPEYAMEGIFSTKSDVYSFGVLLLEVVTG
IRRNS--------------------------------
>HORVU.MOREX.r3.2HG0111810.1_kinase_152_407
LKLVGQGAFGKVFQVRKTSEIYAMKVMRKD-LEHSEMKAERDILTKVDHPFVVQLRY--F
QTKYRLLVLDFVNGGHLFFQLYKQLFREELARIYTAEIISAVAHLHA-NGIMHRDLKPEN
ILL----HAMLTDFGLAKEF-ESMCGTLEYMAPEIIQGQ-HDKAADWWSVGILLFEMLTG
KPPFVDLKL----PPFAHSLLKGLLHKEAGKRLGSDE
>HORVU.MOREX.r3.2HG0130740.1_kinase_64_318
--VLGLGSVKKVYRGFDEGIEVAWNRVRLRARDVERLHAEVRLLRSLHHDHIIGFHKVWD
RDAGVLFITEVCNSGSLREYRDKH-VSLKALKKWARQILEGLDHLHTHPCIIHRDLNCSN
VFINGN-QVKIGDLGLAAI-VDTILGTPEFMAPELYSET-YTESVDIYSYGMCVLEMVTR
EMPYRSVTNGVPPNALMRAFILRCIGK-PRNRPSTAD
>HORVU.MOREX.r3.2HG0132510.1_kinase_32_285
-DVLGKGASKTVYRAFDQGMEVAWNQVKLYDQCLERLYCEIHLLKTLKHKNIMKFYTSWD
VSARHIFITEMFTSGTLRQYRQRK-VNIWAVKDWCRQILSGLLYLHSHPPIIHRDLKCDN
IFVNGN-EVKIGDLGLAAIL--HCVGTPEFMAPEVYAEE-YNELVDIYSFGMCVLEMVTF
EYPYSHVISGTMPEALVRQFVEKCLTT-ASQRLTARE
>HORVU.MOREX.r3.2HG0133380.1_kinase_352_616
ERKLGEGGFGAVYQGLLQNLDVAIKRVAKGSQGRKEYISEVKIISRLRHRNLVQLEGWCH
EHGEFLLVYEFMPNRSLDTHLYNSLLTWPLRFKVTAGVASALLYLHEEQCVIHRDIKPSN

VMLDSVFNAKLGDFGLARLITTVLAGTMGYMAPECVTTGKASKESDVYSFGILALEIACG
RRPVVEAVDSRLDGAALMVVGLWCAHPDYNLRPSIRQ
>HORVU.MOREX.r3.2HG0133550.1_kinase_69_327
GGELGRGEFGVTYLCTDTREALACKSISKK-RTIEDVRREVEIMRHLKHPNIVTLRD--Y
EDDNAVLVMELCEGGELFDRIVARHYTERAAAVVTKTIVEVVQMCHK-HGVMHRDLKPEN
FLFANK-ALKAIDFGLSVFF-PEIVGSPYYMAPEVLKRN-YGQEVDVWSAGVILYILLCG
VPPFWQIDFKRDPPR-AKDLVRGMLNPDPRRRLTAQQ
>HORVU.MOREX.r3.2HG0138550.1_kinase_354_620
SKMLGKGGFGMVYKGVLDGQEIAVKRLCQTSQGIGELKSELVLVAKLHHKNLVRLVGVCL
EEQEKILVYEYMPNRSLDTILFKNELDWGKRFKIINGIARGLQYLHEDLKIVHRDLKASN
ILLDVDYNPKISDFGLAKIFTRRIAGTYGYMAPEYAMRGQYSSKSDVFSFGVLILEIITG
RRNSGNLMDPSLSNHVCIHVGLLCVQRKPASRPTMSS
>HORVU.MOREX.r3.2HG0138610.1_kinase_364_570
SNKLGEGGFGAVYKGVLEGEEIAVKRLSQSSQGIEELKTELVLVAKLQHKNLVRLLGVCL
EGQEKLLVYEYMPNLSLDTVLFKRDLDWGKRLKIVNGVARGLQYLHEDLRIVHRDLKASN
VLLDSDCNPKISDFGLAKLFTSHIAGTYGYMAPEYAMRGQYSVKSDAFSFGVLLLEIVTG
RKNSS-----FASS-----------------------
>HORVU.MOREX.r3.2HG0138720.1_kinase_345_609
KNKLGVGGFGIVYKGILDGQEIAVKKLLGGTSGLHQLHNEVQLLAELQHKNLVRLQGFCS
HRDDTLLVYEYIKNGSLDNFLFEETLSWEQQYNIILGIAKGILYLHEDMRIIHRDLKPNN
ILVDDGMDPKIADFGLARLLTATAVGTLGYMAPEYAIHGRVSPKIDIFSFGVLVLEIVTR
KRNSSEMIDRSLDEHACIHIGLMCVQSDPEDRPYISS
>HORVU.MOREX.r3.2HG0138730.1_kinase_355_560
ANKLGEGGFGAVYKGTLDGEEIAVKRLSKSSQGVKELKNELALVAKLKHKNLVRLVGVCL
EHEERLLVYEFVPNRSLDKILFKRQLDWGKRYKIINGIARGLQYLHEDLKVVHRDLKASN
ILLDTNMNPKISDFGLARLFTSRVVGTYGYMAPEYVMRGNYSVKSDAFSFGVMVLEIVTG
RKNND-----CYN------------------------
>HORVU.MOREX.r3.2HG0138780.1_kinase_40_239
SNKLGQGGFGAVYKGTLDGDEIAVKRLAKSSQGVGELTNELLLVAKLQHKNLVRLVSVCL
EQEERLLVYEFVPNRSLDQVLFKRQLDWGKRYKIISGIARGLQYLHEDLKVVHRDLKASN
VLLDTNMNPKISDFGLARLFTSRVVGTYGYMAPEYVMRGNYSVKSDVFSFGVMVLEIVTG
KK-----------------------------------
>HORVU.MOREX.r3.2HG0138790.1_kinase_350_552
ANKLGEGGFGAVYKGDLDGQEIAVKRLSRHSQGIGELKNELVLVAKLQHKNLVRLVGVCL
QEHEKLLVYEYMPNRSIDTILFRRELDWGKRLKIISGIARGLQYLHEDLRIIHRDLKASN
VLLDFDYTPKISDFGLARLFTNRVVGTYGYMAPEYAMRGHYSIKSDVFSFGILMLEFVTG
RRSSG--------------------------------
>HORVU.MOREX.r3.2HG0138810.1_kinase_359_561
SNKLGEGGFGVVYKGILDNEEIAVKRLSQSSQGIEELKNELVLVAKLQHKNLVRLLGVCL
EEQEKLLAYEYMPNKSLDTILFRSQLDWRMRFRIVNGIARGLQYLHEDLKIIHRDLKSSN
VLLDSEFNPKISDFGLARLFTNRVVGTYGYMAPEYAMRGNYSIKSDVFSFGVLILEIVTG
KRNSV--------------------------------
>HORVU.MOREX.r3.2HG0138840.1_kinase_360_621
RNKLGEGGFGVVYKGILDNEEIAVKRLSQSSQGIEELKNELVLVAKLQHKNLVRLLGVCL
EEQEKLLAYEYMPNKSLDTILFRSQLDWGMRFRIVNGIARGLQYLHEDLKIIHRDLKASN
ILLDSDFNPKISDFGLARLFTNRVVGTYGYMAPEYAMRGSYSIKSDVFSFGVLILEIVTG
KRNSVDIMDSSMTSHMCMHIGLLCVQEDPADRP----
>HORVU.MOREX.r3.2HG0139040.1_kinase_366_633
ENKLGQGGFGPVYKGKFDGAEVAVKRLAAQSQGLVEFKNEIQLIAKLQHTNLVKLVGCCV
QEEEKMLVYEYLPNRSLDFFIFRGLLDWKKRRHIVEGVAQGLLYLHKHVRIIHRDMKASN

ILLDKDLNPKISDFGMARIFTTRVVGTYGYMAPEYASQGLFSVKSDVFSFGVLLLEIVSG
KRNSSHLVDPTLGHCICVKVALLCVQDNAMDRPTMTD
>HORVU.MOREX.r3.2HG0139070.1_kinase_294_562
GKILGQGGFGPVYKGIFDGQEVAIKKLAARSQGLVEFKNEIQLVAKLQHRHLVRLLGCCI
HDEEKILIYEYMSNKSLDYFIFRRSLNWMIRLKIIEGIAQGLLYLHEHLRIIHRDLKASN
ILLDSELNPKISDFGMARIFTSRLVGTYGYMAPEYAFQGLLSIKSDVFSFGVLLLEIISG
KKSAGNFIDQSIGDEMYLRLALMCVQMKAVDRPTMSD
>HORVU.MOREX.r3.2HG0142660.1_kinase_17_267
RDLIGRGSFGDVYRGFDLSKEVAIKVIDLE-EAIEDIQKEISVLSQCRCPYITDYYG--Y
LHQTKLIVMEYMAGGSVADLLQ--PLDEISIACILRDLLHAVEYLHS-EGKIHRDIKAAN
ILLTESG-VKVADFGVSAQL--TFVGTPFWMAPEVIQNSGYNEKADIWSLGITAIEMAKG
EPPLAPIPRENP-PQLMKEFVSLCLRKNPAERPSAKE
>HORVU.MOREX.r3.2HG0155570.1_kinase_368_627
GKLLGSGTFGCVYEATNTGALCAMKEVNIIPKSLKQLEQEIKFLSQFKHENIVQYYG--D
TIEDRFIYLEYVHPGSINKYVKQHAITESVVRNFTRHILRGLAFLHG-QKIMHRDIKGAN
LLVDING-VKLADFGMAKH-LSSLKGTPYWMAPEMVQATGYDLAVDIWSLGCTIIEMFDG
KPPWSGVLHKD--PPIAKDFLQCCFKRNPAERPTASE
>HORVU.MOREX.r3.2HG0169650.1_kinase_292_547
GKLLGSGTFGQVYLGFNNGQFCAIKEVQVISHSLKQLNQEIDMLKKASHPNVVQYYD--E
MTDDTLIYLEYVSGGSIHKLLREYPFKEPVIRNYTGQILAGLAYLHA-KNTVHRDIKGAN
ILVGPNG-VKLADFGMAKH-ISSFKGSPYWMAPEVIMNSGYNLAVDIWSLGCTIIEMATA
RPPWHGIANSKDTPEIGRSFLKLCLKRNPASRATASQ
>HORVU.MOREX.r3.2HG0180480.1_kinase_89_364
HRRIAPGTFGSVYRATYDGKDVLAKLLDWGEIAREALRKEVIVWKELDHPNITKFIGASM
GTDSGEVVVEYLSGGTLRQHLYDK-LTYEAVVELALDLARGLAYLHSK--IVHRDVKAEN
MLLDSKGTLKIADFGVARVQMT-MTGTPGYMAPEVI-LGPYNRKCDVYSFGICLWAIYCC
DMPY-NIVHKNLRPKIMANIMKSCWQADPEKRPDMLD
>HORVU.MOREX.r3.2HG0188270.1_kinase_404_660
GKLIGRGTFGHVYVGFNSGEMCAMKEVTLFSKSAKQLGQEISLLSRLQHPNIVRYYG--E
TVDDKLIYLEFVSGGSIHKLLQEYQLGEPAIRSYTQQILSGLAYLHA-KNTVHRDIKGAN
ILVDPSG-VKLADFGMAKH-INSFKGSPYWMAPEVIKSSGCNLAVDIWSLGCTVLEMATA
KPPWSGIGNSKELPPICKDFIRKCLQRDPSQRPTAME
>HORVU.MOREX.r3.2HG0191490.1_kinase_499_750
KERIGAGSFGTVYRADWHGSDVAVKVLTDQG--LREFLREISIMKRVRHPNVVLFMGAVT
------IVTEYLPRGSLFRLISGEILDLRRRLRMALDVAKGINYLHCLPPIVHWDLKTPN
MLVDKNWSVKVGDFGLSRFKSSSVAGTPEWMAPEFL-RGPSNEKCDVYSFGVILWELLTM
QQP--GVAFQNRRLPILAALVESCWADDPRQRPSFSS
>HORVU.MOREX.r3.2HG0193160.1_kinase_66_318
YEAIGRGKHSTVYKGRKTIEYFAVKSV-----DRSKVLNEVRMLHSLDHANVLKFYS--Y
ETSAHFLVLEYCVGGDLKGLLEQDKLPESAMHDLAYDLVKALLFLHS-QGIIYCDLKPSN
ILL----CMKLCDFGLARRLDPPMRGTPCYMAPELFQEGVHSYASDFWALGCVLYECYAG
RPPFVTPAL----PDNFQNLMDCLLMKDPAERLQWSE
>HORVU.MOREX.r3.2HG0195450.1_kinase_886_1168
GNVIGSSSLSTVYKGVLDGKAVAVKRLNLEQPADKSFLTELATLSRLRHKNLARVVGYAW
EAKMKALVLEYMDNGDLDGAIHPQTVAERLRVCVS--VAHGLVYLHSGFPIVHCDVKPSN
VLLDAHWEARVSDFGTARMLATAFRGTVGYMAPELAYMKSPSPKADVFSFGVMVMELFTK
QRPTGDVLDPGMKVA-ALRLASSCAEFEPADRPDMNA
>HORVU.MOREX.r3.2HG0198460.1_kinase_21_141

------------------------HLGLAQRISIVTNIADALDYLHHYRPIIHCDLKPSN

ILLDIHMNACLGDFGIARFYSSTVNGTLGYMAPEYAENGHASTCGDVYSFGIVLLEMLTG
KRPTD---------------------------------
>HORVU.MOREX.r3.2HG0198470.1_kinase_679_899
INLIGEGSYSSVYKGKLQ-REVAVKVLDLEI--EGSFALECKALRGLRHRNIVPLITECI
DNKFRALIYAFMPNGNLDTWLHQAHLGLAQRISIATNIANALDYLHNDKPIAHCDLKPSN
ILLDIHMNACLGDFGIARFYSNTANGTLGYMAPEYAESGHASTCGDVYSFGIVLLEMLTG
KRPTD---------------------------------
>HORVU.MOREX.r3.2HG0202060.1_kinase_18_271
GDEIGKGAYGRVYKGLDNGDFVAIKQVSL--IPLNIIMQEIDLLKNLNHKNIVKYLG--L
KTNSHLIIILEYVENGSLANIIKKFPFPESLAAVYIAQVLEGLVYLHE-QGVIHRDIKGAN
ILTTKEG-VKLADFGVATKLTESVVGTPYWMAPEVIEMSGVCAASDIWSVGCTVIELLTC
SPPYYPIVQDVQ-PPIITDFLRQCFQKDSIQRPDAKT
>HORVU.MOREX.r3.2HG0202600.1_kinase_86_389
VSTLGEGTYGVVVKARHTGQTVALKTLRSSP--VGELLREACFMAACGHASLVALRGLVP
STNRDYLVMEYV-GPNLRQVFHRGPFPEADVRCIVRQLLAGAETMHR-NGVVHRDIKPEN
ILVDGSGVVKIGDYGLALST--GTAGTRYYMAPEVLLEKDRDTLVDLWSIGCVMAELLTG
KTLFDQVPDDRAFFALGFEVLQGLLTCNPQRRLDAAA
>HORVU.MOREX.r3.2HG0209890.1_kinase_12_151

--KKKN
ILLDDNMIPKIVDFGLSRLLTSSL-GTFGYMAPEFVDRGTITPKSDIFSLGVIIIEIIMG
HRDYPDRLARSQR--ICVQIGLICVNPDRTKRPPARN
>HORVU.MOREX.r3.2HG0214180.1_kinase_2_257
---------------------IAVKRIISPF--QKQFNSEVCHLMLLKHPNIVTFVGYCC
ETKEMLLCFEYLPNGSLDKYLSESGLDWSTRYKIIEGICYGLCHLHEQRHIVHLDLKPAN
ILLDGSMVPKIADFGLSRLLTSSRDGTLGYMAPEFLHGGTITQKSDIFSLGVIIMGVVTG
HRDYPEVLHRSLG--ICIKTSLICVNPERNKRPRIMK
>HORVU.MOREX.r3.2HG0214800.1_kinase_28_275
--------ATSVWRAVRTGAPAAVKQVRLA-PADS-LDCELRFLAAVSHPNIIRLLD--I
RTPGCILVMELCEGGDLASYIERSRVDESVARNFMRQIGAGLQVLRR-HHVVHRDLKPEN
ILLSCR-MLKISDFGLSRVL-PTACGTRLYMAPEVMLFQ-YDDKVDLWSIGAILFELLNG
YPPFRVLPFSQLVPS-SIDICTRLLCTNPVKRLSLQE
>HORVU.MOREX.r3.2HG0214820.1_kinase_275_519
KEKIASGSSGDLYRGTYLDVDVAIKFLRTEH--KVEFLQEIMILRSVNHENVVRFYGACT
------IVTEYMAGGNLYDFLH-T-LELSLILRIAIGISKGMDYLHQN--IIHRDLKSAN
LLIGDGQVVKIADFGVSRQRMT-ETGTYRWMAPEVI-NHPYDHKADVFSFAIVLWELVTS
KVP--NVRQ-GLRLVIISKLIQRCWGENPHTRPVFSE
>HORVU.MOREX.r3.2HG0215640.1_kinase_349_614
RNKLGEGGFRVVYKGILDGQEIAVKKLRGRAHGLHQLHNEVLVLAELQHKNLVQLRGFYS
HRDDTLLVYEYIKNGSLARYLSEGTLIWEQKYNIILGVAKGILYLHEDIRIIHRDLKPNN
ILLDEDMEPKIADFGLARLLTSRAVGTLGYMAPEYVYNRRVSPKIDIFSYGLLILQIVTT
RRKCWDMMDKTLNQHQCIYVGLMCVQADPSDRPEIST
>HORVU.MOREX.r3.3HG0218320.1_kinase_70_313
-----SGRHSRVYFGRYNGREVAIKMVSQPHAAERQFASEVALLLRLRHHNIVSFVAACK
------IITEYMAGGSLRKYLHHS-VPIQLVLQLALDIARGMSYLHSQ--ILHRDLKSEN
VLLGEDMSVKVADFGISCLEGK-FTGTYRWMAPEMI-KENHTRKVDVYSFGIVLWEILTA
LVP--EVALKNARPPLMSHLISQCWATNPDKRPQFDD
>HORVU.MOREX.r3.3HG0219050.1_kinase_341_544
QNLLGEGGFGSVYKGCLEGREVAIKKLKDGSQGEREFQAEVEIISRVHHRHLVSLVGYCI
SGDQRLLVYDFVPNDTLHYHLHGVVLDWPARVKISAGSARGIAYLHEDPRIIHRDIKSSN

ILVDNNFEAQVADFGLARLATTRVMGTFGYMAPEYASSGKLTEKSDVFSFGVVLLELITG
RKPVD--------------------------------
>HORVU.MOREX.r3.3HG0221130.1_kinase_355_568
ENKLGHGGFGAVYKGVLHGRQIAVKRLDRASQGVKELRNELLLVAKLRHNNLTKLLGVCL
KGKEKLVVYEYLPNRSLDIFLFKRLLPWETRYRIIYGTARGLLYLHEDVRILHRDLKASN
ILLDTDMNPKISDFGLARLFTSQVVGTLGYMAPEYAVRGRMSVKIDVYSFGVLVLEIVTG
RKNTD-LEESSLED-----------------------
>HORVU.MOREX.r3.3HG0222300.1_kinase_59_266
RNLLGRGGFGPVYKGVMGGQEVAVKRLSLESQGVREFLNEVRLLLKVQHRNLVSLLGCCA
AAGQKMLVYPYFPNGSLDHILFKRQLDWPKRHQIILGLARGLLYLHEEVKIIHRDIKASN
VLLDEKLNPKISDFGMARLFTFRISGTYGYMAPEYAMNGYLSTKTDVFSFGMLVLEIVSG
RKN----IDRHLGDE----------------------
>HORVU.MOREX.r3.3HG0225230.1_kinase_327_533
ESKLGQGGFGAVYKGHLDGSEIAVKRLASHSQGFMEFKNEVQLIAKLQHTNLVRLLGCCS
QEEEKILVYEYLPNKSLDFFIFKRLLDWTKLLAIIEGVAHGLLYLHKHLLVIHRDLKPSN
ILLDSEMNPKISDFGLAKIFTRRVVGTYGYMAPEYASKGIFSIKSDVFSFGVIIFEILSG
KRNSGQ-------------------------------
>HORVU.MOREX.r3.3HG0225240.1_kinase_393_596
ENKLGEGGFGPVYKGRFDGVEIAVKRLDSDSQGFIEFKNEVELIAKLQHRNLVRLMGCCS
QGEEKILVYEYLPNKSLDFFIFRKQLDWDKRIVIILGTAEGLLYLHKHLRVIHRDLKPSN
ILLDSQMNAKISDFGLAKIFTRKVVGTYGYMAPEYASHGIFSVKSDVFSFGVLTLEIVSG
KRNSH--------------------------------
>HORVU.MOREX.r3.3HG0225250.1_kinase_23_205
------------------GLEIAVKRLASHSQGFLEFKNEVQLIAKLQHTNLVRFLGCCS
QEEEKILVYEYLANKSLDIFIFKRLLDWSKLLEIIEGIAHGLLYLHKHLLVIHRDLKPSN
ILLDSEMNPKISDFGLAKILTRRVVGTYGYMAPEYASKGVFSIKSDVFSFGVVIFEILSG
KRNSG--------------------------------
>HORVU.MOREX.r3.3HG0225260.1_kinase_367_570
ENKLGQGGFGPVYKGQFDGNEIAVKRLDSRSQGFREFKNEVELIAKLQHRNLVRLMGCCS
QGEEKILVYEYLPNKSLDFFIFRKLLDWDKRLAIIVGIAEGLLYLHKHLRVIHRDLKPSN
VLLDSEMNAKISDFGLAKIFTRKVVGTFGYMAPEYASHGIFSIKSDVFSFGVLTLEILSG
KRNSH--------------------------------
>HORVU.MOREX.r3.3HG0225270.1_kinase_368_632
ENKLGEGGFGPVYKGRFDGVEIAVKRLDSDSQGFIEFKNEVELIAKLQHRNLVRLMGCCS
QGEEKILVYEFLPNKSLDFFIFRKLLDWDKRIAIIVGTAEGLLYLHKHLRVIHRDLKPSN
ILLDSEMNAKISDFGLAKIFTRKVVGTYGYMAPEYASHGIFSIKSDVFSFGVLTLEIVSG
KRNSHELIDAALLPDMCINIALLCVQENAADRPTMLD
>HORVU.MOREX.r3.3HG0225280.1_kinase_326_528
ESKLGQGGFGAVYKGQLDGLEIAVKRLASHSQGFMEFKNEVQLIAKLQHTNLVRLLGCCS
QEEEKILVYEYLPNKSLDFFIFKRLLDWTEIVAIIEGVANGLLYLHKHLLVIHRDLKPSN
ILLDSEMIPKISDFGLAKIFTRRVVGTYGYMAPEYASKGNFSIKSDVFSFGVVILEILSG
KRNS---------------------------------
>HORVU.MOREX.r3.3HG0234500.1_kinase_330_527
ANKLGQGSNGAVYKAVMDGKEVAVKRLFLNTEWVEQFFNEVELISQVRHKNLVKLLGCSV
NAPESLLVYEYYFNKSLELFLFRSKLTWGLRVGIIQGIAEGLSYLHEETRIIHRDIKASN
ILLDDKYKPKITDFGLARAFTTGVAGTLGYMAPEYVVHGHLTEKADVFSYGILVLEIVTG
KRC----------------------------------
>HORVU.MOREX.r3.3HG0243960.1_kinase_323_570
-----SGAHSRLYRGLYDDKPVAIKFIRRPHAADKQYNTEINALSHLYHKNVIKLVATHK
------IITELLPGGSLRSYLHRP-LPLERTISIALEIARGLEYVHSQ--VVHRDIKPEN

ILFDENFEVKIADFGIACEELL-DEGTYRWMAPEMI-KRPYNRKVDVYSFGLLLWEMVTG
RIP--NVATDKLKPMDVRPLIEQCCALQPEKRPDFWQ
>HORVU.MOREX.r3.3HG0254050.1_kinase_4_256
YEAIGRGKHSTVYKGRKTIEYFAVKSV-----DRSKVLNEVRMLHSLDHPNVLKFFS--Y
ETSAHFLVLEYCVGGDLKGLLEQDKLPESAMHDLAYDLVKALLFLHS-QGIIYCDLKPSN
ILL----CMKLCDFGLARRLDPPMRGTPCYMAPELFQEGVHSYASDFWALGCVLYECYAG
RPPFVTPPL----PDNFQNLINCLLMKDPAERLQWSE
>HORVU.MOREX.r3.3HG0264760.1_kinase_36_312
LSPIARGSESTVYEARLGGRAAAKKPVLSTS--LDKFHYQLQLLCELDHPGLAKLIAAHA
RP-NYLMFFEFFEPPNLADKIHEESPSIQQVVMIASYLAKTLQYLQIF--IVHRDIKPAN
ILLDKDLLPHLADFGLAMYQSSNMVGTLIYMAPEILRKGLHTEKSDVYSFAISINELLTG
VVP--DVVSQGLRPALLLSLIQRSWDSDPERRPSFGD
>HORVU.MOREX.r3.3HG0274630.1_kinase_355_558
DKVIGEGGFGKVYMGALDGRRVAVKQLKVGGQGEKEFRAEVDIISRIHHRHLVTLVGYCV
TENHRLLVYEFVSNDTLEHHLHGLVMDWPKRMKIAIGSARGLTYLHEDPRIIHRDIKSAN
ILLDDAFEAKVADFGLAKLTSTRVMGTFGYMAPEYASSGKLTDRSDVFSFGVVLLELITG
RKPVD--------------------------------
>HORVU.MOREX.r3.3HG0274900.1_kinase_84_353
KGVIARGTFGTVHRGVYDGQDVAVKLLDWGEITRSAFAQEVAVWHKLDHPNVTKFIGAIM
GADHGHVVVEYLAGGALKNFLIRK-LAFKVVVQLALDLARGLSYLHSE--IVHRDVKTEN
MLLDKTRTVKIADFGVARVEMT-ETGTLGYMAPEVL-NGPYNRKCDVYSFGICLWEIYCC
DMP--DVVRQNLRPEILANVMKRCWDANPDKRPEMAE
>HORVU.MOREX.r3.3HG0278430.1_kinase_8_229

--DDRLLVMEYV-GPSLHHVLHRRPFQEDVVRYLMRQLLGGAKHMHE-CGVVHRDIKPEN
VLV----GVKICDFGLAMRT--GLHGTRSYMAPEILLGKDYDAKVDAWSLGCVMAEILLG
DRLFGDVPDQISWPSYGFQVLSGLLSCDAHKRLSAAD
>HORVU.MOREX.r3.3HG0279020.1_kinase_512_795
KEELGKGGNGVVYRGILDKKVVAVKKLTDVRKGEEEFWAEVTLIGRINHMNLVRMYGFCS
EGQHRLLVYEFVENESLDRYLFTELLSWGQRFKIALGTARGLAYLHHEEWIVHCDVKPEN
ILLTREFEAKIADFGLSKLSFTQMRGTTGYMAPEWVMNLPIDAKVDVYSFGVVLLEIVTG
SRVSSDIVDARLKGHAMVKIAISCL-DERSKRPTMHQ
>HORVU.MOREX.r3.3HG0279030.1_kinase_508_716
KEEIGRGGAGIVYRGVLDKKIVAVKKLTNVQQGEEEFWAEVTLIGRINHINLVRMMGFCS
EGKNRLLVYEYVENESLDKYLFTELLGWNQRYKIAVGAARGLAYLHHEEWIVHCDVKPEN
ILLTRDFDAKIADFGLAKLAFTHMRGTMGYMAPEWALNTPINAKVDVYSYGVVLLEIVTG
ARVSSD-------------------------------
>HORVU.MOREX.r3.3HG0279070.1_kinase_494_764
KEEIGRGGSGIVYRGVLDKRVVAVKNLTSVSHSEEEFWAEMNIIGRINHMNLVRMWGFCS
EGQHKLLVYEYVENESLDKFIFAELLAWSQRFKIALGTARGLAYLHHEEWVIHCDIKPEN
ILLTRDFEAKIADFGLAKLSLTHMRGTMGYMAPEWALNLPIDAKVDVYSYGVVLLEIVTG
NRISSDIADARLNGHVMMKLALSCL-EERNSRPTMNE
>HORVU.MOREX.r3.3HG0279100.1_kinase_541_816
NEELGRGGSGVVYRGVLKTTVVAVKRLTNVVQGEEEFWAEMTVFGRINHINLVRIWGFCS
EGQHKLLVYEYVENESLDRHLFMGSLAWSERFKIALGVARGLAYLHHEEWVIHCDVKPEN
ILLTRDLDAKIADFGLAKLSLSHMRGTAGYMAPEWALGLPVDAKVDVYSYGIVLLEIVIG
SRISDDLVDSRLNGQAMVKISLSCM-EERNNRPTMDD
>HORVU.MOREX.r3.3HG0279390.1_kinase_544_649
GTRVGIGFFGEVFRGIWNGTDVAIKVFLEQD--MEDFCNEIYILSRLRHPNVILFLGACM
------MVTEYMEMGSLYYLIHKKKLSWRRRLKIIRDIC---------------------

>HORVU.MOREX.r3.3HG0281980.1_kinase_796_996
DNLLGAGGFGKVFKGQLDESIIAIKVLNMQDVA-KTFDTECRALRMARHRNLVRIISTCS
NLDFKALVLEYMPNGSLDDWLHGRHISFLQRLGIMLDVAMAMEYLHHREVVLHLDLKPSN
ILLDMDMTAHVADFGISKLLLTSMPGTVGYMAPEFGSTGKASRKSDVYSFGIVLLEVFTR
KKPTD--------------------------------
>HORVU.MOREX.r3.3HG0282860.1_kinase_10_257
LRTLGCGASGAVSLAADSGELFAVKS-----GAAAQLSREQGILSGLCSPHVVACIGGGG
ARDGSYLFLEFAPGGSLADEVARNRLEERAVRAYAADVLRGLAYIHG-ESVVHGDVKPRN
VVIGADG-AKMADFGCARA-VGG--GTPAFMAPEVARGEEQGPAADVWALGCTVVEMATG
RAPWS-IGYTDAVPEVAKSFLAACFARSARDRCTAAQ
>HORVU.MOREX.r3.3HG0282880.1_kinase_71_317
VRTLGRGASGAVSLAADSGALFAVKS-----AAADQLRREGDILSGLCSPHVLPCLGFRA
GAAGECLFLEFAPGGSVADVAERSRLEECAIRPYAADVARGLAYLHG-RSLVHGDVKARN
IVVGADG-AKIADFGCART-AGG--GTPAFMAPEVARGEEQGPAADVWALGCTVIEMATG
RAPWS-IGYTDAVPAVAKHFLSMCLARNARDRCTSAQ
>HORVU.MOREX.r3.3HG0282920.1_kinase_11_257
LRTLGRGASGAVWLASDSGQLLAVKS-----AGADTLRREGRVMAGLCSPHVVPCLGSRA
AAGGEYLFLEFAPRGSLADEAARSSLAERAIQGYAADVASGLAYLHG-NSLVHGDVKARN
VMVGADG-AKLADFGCARA-AGA--GTPAFMAPEVARGEEQGRAADVWALGCTVIEMATG
RAPWG-IGYTDAVPELAKDFLRKCLARNPRHRPTAAE
>HORVU.MOREX.r3.3HG0288480.1_kinase_298_544
-----SGAHSRLFHGIYKEAPVAVKLIRQPDASEKQFNTEIVTLYRLHHRNVIKLIGACR
------VITEFLSGGSLRAFLHRS-LPLDKIISVGLDIAHGMGYIHSQ--IVHRDVKPEN
IIFDRDCCAKIVDFGIACEELA-DPGTFRWMAPEMM-KHPYGRKVDVYSFGLILWEMLTG
SVP--DVFDKNVRPPILRVLIEQCWTLQADKRPEFWQ
>HORVU.MOREX.r3.3HG0293940.1_kinase_498_764
ENKLGEGGFGPVYKGNLNGQDVAVKRLAANSQGLPEFKNEILLIAKLQHSNLVGLLGCCI
DGEEMLLIYEYMPNKSLDFFLFRRFLVWAMRLNIIEGIAQGLIYLHKHLRIIHRDLKPSN
ILLDTDMNPKISDFGMARIFTKRVVGTYGYMAPEYAMAGIFSVKSDVYSYGVLLLEIISG
LRNAA-LIDKSLHGAVCIHVGLLCVQENAADRPSMAE
>HORVU.MOREX.r3.3HG0293970.1_kinase_516_781
ENKLGEGGFGPVYKGNLNGQDVAIKRLAANSQGLPEFKNEILLIAKLQHTNLVGLLGCCI
DGEEMLLIYEYMSNKSLDFFLFRRILVWEMRLNIIEGIAQGLIYLHKHLRVIHRDLKPSN
ILLDNDMNPKISDFGMARIFTKRVVGTYGYMAPEYAMAGIFSVKSDVYSYGVLLLEIISG
LRNAA-LIDKYLHGAVCIHVGLLCVQENAADRPSMAE
>HORVU.MOREX.r3.3HG0294080.1_kinase_521_605
DNKLGEGGFGPVYKGKLDGQEIAVKTLSKTSQGLDEFKNEVMLIAKLQHRNLVRLLGYSI
SGHERLLVYEYMENKSLDYFL---------------------------------------

>HORVU.MOREX.r3.3HG0303750.1_kinase_75_331
DRELGRGEFGVTYLCVDTREQLACKSISKR-RTVEDVRREVAIMRHLRSHSIVALRE--C
EDEGAVLVMELCEGGELFDRIVARHYTERAAANVTRTIVEVVQLCHR-HGVIHRDLKPEN
FLFANK-PLKAIDFGLSIFF-PEIVGSPYYMAPEVLKRN-YGPEIDIWSAGVILYILLCG
VPPFWQIDFKREPPN-AKDLVRRMLEPDPKLRLTAKQ
>HORVU.MOREX.r3.3HG0309360.1_kinase_435_683
GEQVGQGCCGTVYHALWHGSDVAAKVFSKQE--INTFRQEVSLMKKLRHPNIILFMGAVL
------IVTEFLPRGSLFRLLRK--LDPRRRVNMAIDIARGMSYLHNSPTVVHRDLKSPN

LLVDKNWTVKVADFGLSRLKTTTGKGTPQWMAPEVL-RSPSNEKSDVFSYGVVLWELVTQ
NIP--TVGFMDHRLEIWASMIQSCWDSDPQRRPSFQE
>HORVU.MOREX.r3.3HG0309380.1_kinase_429_677
GEQVGQGCCGTVYHALWHGSDVAAKVFSKQE--INTFRQEVSLMKKLRHPNVILFMGAVL
------IVTEFLPRGSLFRLLRK--LDPRRRVNMAIDIARGMNYLHNSPTVVHRDLKSPN
LLVDKNWTVKVADFGLSRLKTTTGKGTPQWMAPEVL-RSPSNEKSDVFSYGVVLWELVTQ
KIP--TVGFMDDRLEILASMIQSCWDSDPQCRPSFQE
>HORVU.MOREX.r3.3HG0314450.1_kinase_109_335
EHALGSGGSGVVYKGRMNGEVIAVKKL-QPS--QRLFENEVYRLMYLNHPHIVRLRGYCY
ETQERLLCLQYYPKGSLAGYISESGLKWDTRYNIIVGICYALKYLHEEKPVLHMDLKPAN
ILLDDAMRAKITDFGLARLLITSRDGTLGYMAPEYLHGGKVTTKSDIFSLGAIILEVITG
HKDYTR-------------------------------
>HORVU.MOREX.r3.3HG0322220.1_kinase_3_64

--------PKISDFGLAKNFTKRVVGTYGYMAPEYASEGIYSIKSDVFSFGVLLLEILSG
KR-----------------------------------
>HORVU.MOREX.r3.3HG0322270.1_kinase_385_652
ENLLGQGGFGPVYKGQLDGTEIAVKRLASHSQGFTEFKNEVELIAKLQHSNLVKLMGCCI
KGEEKLLVYEYLPNKSLDFFIFRTLVDWNKRCEIIEGIAQGLLYLHKHLRIIHRDLKASN
ILLDQDMNPKISDFGLAKIFTKKVVGTYGYMAPEYASEGIYSTKSDVFSFGVLLLEILSG
KRNSGQLLEASIAEEAYIHIALMCVQEHADDRPTMSN
>HORVU.MOREX.r3.4HG0332630.1_kinase_504_754
KEKIGAGSFGTVHRADWHGSDVAVKILMEQD--FKEFMREVAIMKSLRHPNIVLFMGAVT
------IVTEYLSRGSLYKLLHREVLDERRRLNMAFDVAKGMNYLHRRPPIVHRDLKSPN
LLVDKKYTVKVCDFGLSRLKSSSLAGTPEWMAPEVL-RDPSNEKSDVYSFAVILWELMTL
QQP--NVGFKGRRLEIVAALIESCWINEPWRRPSFAN
>HORVU.MOREX.r3.4HG0334900.1_kinase_91_350
GRELGRGEFGVTRLATDARERLACKSIPKA-RTVADVRREVAIMASLDHPALVRMRA--Y
EDDDAVLVMELCDGGELFDRIVARRYTERAAAAAARTVAEVVRACHA-HGVMHRDLKPEN
FLYAGK-QLKAIDFGLSVFF-PEIVGSPYYMAPEVLRRS-YGPEVDIWSAGVILYILLCG
VPPFWQLDLDREPPR-AKSLVRQMLHMDPRKRLTARQ
>HORVU.MOREX.r3.4HG0339400.1_kinase_521_746
-DEVGHGAYGTVFRGELDRRAVAVKQL----SGEAEFWAEVTIIARMHHLNLVRMWGFCA
DKDKRMLVYEYVPNGSLDKYLFQKMLDLHTRYRIALGVARAIAYLHEEEWVLHCDIKPEN
ILLEDDFCPKVSDFGLSKLTMSRIRGTRGYMAPEWVIHRPITAKADVYSFGMVLLEIVSG
RRNYG--------------------------------
>HORVU.MOREX.r3.4HG0345000.1_kinase_1142_1400
RKLIGSGTFGCVYEATNTGAVCAVKEINIIPRSLKQLDQEIKLLSQFKHENIVQYYG--E
TIKDQLVYMEYVYPGSINKYIKQHAITESIVCNFTRHILRGLAFLHG-QNIMHRDIKGEN
MLIDVNG-VKLADIGMAKH-LCSLKGTPYWMAPEMVRASGYDLAVDIWSLGCTIIEMFNG
KPPWSGVLNKD--PPLAKDFLECCFKRDLAVRPSASQ
>HORVU.MOREX.r3.4HG0346120.1_kinase_247_502
GKLLGSGTFGQVYLGFNGGQMCAIKEVKVIANSLRQLNQEMLLLNQLSHPNIVQYYG--E
LSSETLVYLEFVSGGSIHKLLQEYPFGEAVLRSYTAQILSGLAYLHG-RNTVHRDIKGAN
ILVDPNG-IKLADFGMAKH-ISSFKGSPYWMAPEVIMNTGYSLSVDIWSLGCTILEMATA
RPPWSGIGNSKDIPDIAKSFLKLCLQRDPAARPTAAQ
>HORVU.MOREX.r3.4HG0349400.1_kinase_94_368
DNIIGDGSFGFVYRAVLDGPAVAVKRLSADHAGNREFRAELEVLGSLSHRNLARLLGYCA
AGRDRLLVYELLERGSLDAWLHASPLPWPARLRVTRGAAAALAFLHHDPPVLHRDVKSSN

VLLDEGFEAKLADFGHARVVSTQAAGTAGYMAPEIREGVGASVKADVYSFGVLMMETVTG
RRPSWEIADHRMGLEVFLDIAQSCTEESPKYRPTMRE
>HORVU.MOREX.r3.4HG0356280.1_kinase_534_806
KHELGWGGSGIVYKGTLDEREVVIKRLENVTQNRAEFQDELHVIARINHMNLARIWGVCS
ERSHRMLVLEYFENGSLANILFNKSLLWDQRFNIALGVAKGLAYLHHEEWVIHCNLKPEN
ILLDQDLEPKITDFGFAKLLVSRARGTLGYMAPEWVTGLPITAKVDLYSYGIVLLELVSG
TRILDDFVDIRLDGDTLIRIAVSCLEEDRKNRPTMES
>HORVU.MOREX.r3.4HG0356290.1_kinase_346_620
-AEIGHGGSGTVYKGVLDDRTVAVKVLQDVSQSEEVFQAELSAIGRIYHMNLVRMWGFCS
EGAHRILVYEYVHNGSLANALF--LLGWKQRFNIAVGVAKGLAYLHNEEWIIHCDMKPEN
ILLDDEMEPKITDFGLAKLLLSRIRGTRGYMAPEWVSSLPITDKVDVYSYGVLLLELMKG
KRVSDDLVDERLDGQAFAQLAVSCLEEDRNKRPGMKS
>HORVU.MOREX.r3.4HG0356360.1_kinase_569_851
-DEIGRGGSGVVYRGVLDGRVVAVKALTTSVHGEEEFQAELIVIGRIYHMNLVRIIGCCS
PGKHRILVSEFIENGSLATMLFDHVLGWSQRFRIAVGVARGLAYLHSEEWIIHCDMKPEN
ILLDRDLEPKITDFGLAKLLPSRIRGTRGYMAPEWVSSLAISDKVDVYSFGVVLLELVKG
VRVADDLVDDRLAGDVVVGVALSCLEEERNRRPSMSA
>HORVU.MOREX.r3.4HG0359590.1_kinase_461_709
GEQVGEGSCGTVYHALWYGSDVAVKVFSRQE--IRTFRQEVSLMKKLRHPNIILFMGAVA
------IVTEFLPRGSLFRLLRK--LDPRRRVHMAIDIARGMNYLHSSPTIVHRDLKSSN
LLVDKNWTVKVADFGLSRLKTTTGKGTPQWMAPEVL-RNPSNEKSDVYSYGVVLWELVTQ
KIP--TVGFMDHRLEIWASMIESCWDSDPQRRPSFQE
>HORVU.MOREX.r3.4HG0378900.1_kinase_152_408
LKLVGQGAFGKVYQVRRTSEIYAMKVMRKD-LEHAEMKAERDILTKVDHPFVVQLRY--F
QTKYRLLVLDFVNGGHLFFQLYQQLFREELARLYTAEIVSAVAHLHA-NGIMHRDLKPEN
ILL----HAMLTDFGLAKEF-ESMCGTVEYMAPEIVQGR-HDKAADWWSVGILLFEMLTG
KPPFFDMKL----PTYVHSLLKGLLHKEAGRRLGSDE
>HORVU.MOREX.r3.4HG0384620.1_kinase_6_263
GKCIGKGAFGTVHLAVHTGRAFAVKSVDAKGAAMACLETEIRILRRLCSPYVVAYLG--D
ATAASRLHMELVSGGCAAD----A-LGERAARRVLRRVAAALHYLHDVAGVVHGDVKGRN
VLVGGGGGSKLADFGAARL-VSGPRGTPAWMAPEVARGGASTPASDVWSLGCTAVELLTG
KRPWSGIGYGGKRPELCNDFLDKCLRRDAGERWSCEQ
>HORVU.MOREX.r3.4HG0389130.1_kinase_17_273
LRPIGSGAYSQVWLGKHRGTEVAVKEIAMD-SADS-LLSEVDILRRITHPNIIALHD--I
RDGGRILILEYCRGGDLYAYLLRHRVPETVAKHFIRQLACGLQKLRE-SNVVHRDLKPQN
ILLVSN-ILKIADFGFAKFL-PTLCGSPLYMAPEVMQAQ-YDAKADLWSVGIILYQLVTG
SPPFNILRF----PSDCIDLCRKLLRISSVERLTVEE
>HORVU.MOREX.r3.4HG0390270.1_kinase_319_570
GVLLGSGSFGTVYEGI-EGVFFAVKEVSLHDNAIFQLEQEIALLSQFEHENIVHYFG--D
KEDSKLIFLELVTQGSLVSLYQKYRLRDTHVSAYTRQILNGLTYLHE-RNIVHRDIKCAN
ILVHANG-VKLADFGLAKQ-TSSCKGTVYWMAPEVVNPKTYGPAADIWSLGCTVLEMLTR
QLPYPWIGKGEP-PAIARDFISQCVKPNPEDRPSASK
>HORVU.MOREX.r3.4HG0397630.1_kinase_43_347
VACLGKGGFGVVHRMRHTKKNVAVKFLSSPD--VKDLEQEARFLEACGNPYVVGFEGLV-
--DGDTLVMEYVEASSLRSLLSRDPLPESTVCDFMWKLLTGADKMHEHRHIVHRDIKPAN
ILVGKNLLLKICDLGLAMFM--NRAGTTSYMAPEMILGKDYDMLVDTWSIGCVFAELLTG
KTLFKDMPDERTWPEFGFQVLQGLLTCNPDERLTAAD
>HORVU.MOREX.r3.4HG0398620.1_kinase_43_347
VECLGKGGFGVVLRVRHTKKDLAVKFLSSPD--VEDLHREAGFLEACGNPYVVGFEGMV-
--DGDVLAMEYVEGSSLESLLWRDPLPESMVRDFMWKLLNGAEKMHD-RHVVHRDIKPGN

ILVGETGLVKICDLGLAISM--NQAGTSSYMAPEMIMGKDYDALVDSWSIGCVFAELLTG
ATLFMEVPDERTWPGCGFQVLRGLLTCNPDKRLTAAA
>HORVU.MOREX.r3.4HG0398730.1_kinase_43_349
VACLGKGGFGVVHRMRHTKKNVAVKFLSSPD--VKDLEQEARFLEACGNPYVVGFEGLV-
--DGDTLVMEYVEASSLWSLLWRDPLPESTVRDFMWKLLTGADKMHEHCHIVHRDIKPAN
ILVGKNLLLKICDLGLAMSM--NRAGTASYMAPEMILRKDYEAVVDTWSIGCVFAEVLTG
KTLFNDMSDERTWPEFGFQVLQGLLTCNPNERLTAAD
>HORVU.MOREX.r3.4HG0398740.1_kinase_43_349
VACLGKGGFGVVHRMRHTKKNVAVKFLSSPD--VKDLEQEARFLEACGNPYVVGFEGLV-
--DGDTLVMEYVEASSLWSLLWRDPLPESTVRDFMWKLLTGADKMHEHRHIVHRDIKPAN
ILVGKNLLLKICDLGLAMSM--NRAGTASYMAPEMILGKDYEAVVDTWSIGCVFAELLTG
KTLFNDMPDERTWPEFGFQVLQGLLTCNPNERLTAAD
>HORVU.MOREX.r3.4HG0398750.1_kinase_43_349
VACLGKGGFGVVHRMRHTKKNVAVKFLSSPD--VKDLEQEARFLEACGNPYVVGFEGLV-
--DGDTLVMEYVEASSLWSLLWRDPLPESTVRDFMWKLLTGADKMHEHRHIVHRDIKPAN
ILVGKNLLLKICDLGLAMSM--NRAGTASYMAPEMILGKDYEAVVDTWSIGCVFAELLTG
ETLFMEMPDERTWPEFGFQVLQGLLTCNPDERLTAAD
>HORVU.MOREX.r3.4HG0398760.1_kinase_43_349
VACLGKGGFGVVHRMRHTKKNVAVKFLSSPD--VKDLEQEARFLEACGNPYVVGFEGLV-
--DGDTLVMEYVEASSLWSLLWRDPLPESTVRDFMWKLLTGADKMHEHRHIVHRDIKPAN
ILVGKNLLLKICDLGLAMSM--NRAGTASYMAPEMILGKDYEAVVDTWSIGCVFAELLTG
KTLFNDMPDERTWPEFGFQVLQGLLTCNPDERLTAAD
>HORVU.MOREX.r3.4HG0401890.1_kinase_733_980
GERIGLGSYGEVYRADWNGTEVAVKKFLDQD--LDEFRSEVRIMRRLRHPNIVLFMGAVT
------IVSEYLPRGSLYKILHQ--IDEKRRIKMAIDVAKGMNCLHTSPTIVHRDLKSPN
LLVDNNWNVKVCDFGLSRLKSSSTAGTPEWMAPEVL-RNQSNEKCDVYSFGVILWELATL
RMP--GVGFQDRRLDIVARIIWECWQKDPNLRPSFAQ
>HORVU.MOREX.r3.4HG0403960.1_kinase_353_561
SGLLGSGGFGHVYKGVLSGEEVAIKRISSGTQGMREFVAEVSSLGRMHHRNLVELRGWCK
HGQDLLLVYEFMPNGSLDEHLF--VLTWAQRFSVIRGVARGLLYLHEEHVVVHRDVKANN
VLLGADMGARLGDFGLARLYTTRVAGTLGYMAPELTVTSRATTATDVFSFGALLLEVACG
RRPVED-------------------------------
>HORVU.MOREX.r3.4HG0408170.1_kinase_73_330
IQVIGKGSGGVVQLVRHVGTFYALKGIQMN-IQRKQIVQELKINQATQSPHIVLCHQ--F
YHNGVILVLEYMDRGSLADIIKKT-ILEPYLAVLCKQVLEGLLYLHHERHVIHRDIKPSN
LLVNHKG-VKITDFGVSAVLASTFVGTYNYMAPERISGSSYDYKSDVWSLGLVILECAIG
RFPYTGAIVDQPPPSAFCSFISSCIQKDPAERKSASE
>HORVU.MOREX.r3.4HG0411380.1_kinase_536_801
SHMIGQGGFGKVYKAVLDGREVAIKRLSRNSQGMTEFRNEVVLIAKLQHRNLVSLVGCCS
EGDEKLLIYEYMPNKSLDALLFGEMLDWPTRFRIIKGVAKGLLYLHQDLKIIHRDLKASN
VLLDEEMRPKIADFGMARMFTKRVVGTYGYMAPEYAMRGIFSTKSDVYSFGVLTLEVVSG
VKISSRLVDSNIVGTACVQMGLLCVQDNPNDRPTMSY
>HORVU.MOREX.r3.4HG0416700.1_kinase_30_291
MEEVGFGANAVVYRAIFANRTIAVKCLDLD-RVLDDVRKEAQIMSLIDHPNVIRAYC--F
VVDHNLVIMPFMAEGSCLHLMKAHGLEEPVICSILKETLKALAYLHG-QGHIHRDVKAGN
ILIDSPG-VKLGDFGVSACL-DTFVGTPCWMAPEVLQPGGYNFKADIWSFGITALELAHG
HAPFSP-TLQNAPPGLFKEMVAMCLVKDQTKRPTAEK
>HORVU.MOREX.r3.5HG0421130.1_kinase_377_644
SRKLGEGGSGSVYDGRL-GERVAVKLLDRHAHRQKEFSAEVQTIGSIHHINLVKMIGFCA
DKTNRLLVYEYMSGGSLDKWIYSNPLGWCVRRRIIMHIARGLYYLHEGQRIAHLDIKPQN

ILLDDNFNAKVADFGLSRLIVTRMRGTPGYMAPEW-LTSKITEKVDVYSFGIVVIEILSG
RRNIDEMIDGNNQDMVIMKLAMWCLQSDSNRRPSMSV
>HORVU.MOREX.r3.5HG0421310.1_kinase_684_920
ENLIGSGGSGNVYRVKL-GTVVAVKHITRTRSACREFDAEVGTLSSIRHVNVVKLLCSVT
SEDASLLVYEHLPNGSLYERLHKLGLGWPERYEVAVGAARGLEYLHHGRPILHRDVKSSN
ILLDEAFKPRIADFGLAKILSSAVAGTVGYMAPEYAYTRKVTEKSDVYSFGVVLMELATG
RAAVAD-------------------------------
>HORVU.MOREX.r3.5HG0437110.1_kinase_848_1107
LRELGSGTFGTVYHGKWRGSDVAIKRIKKSCQETKDFWREAQILSKLHHPNVVAFYGVVP
------TVAEFMVNGSLRNVLLS--LDRRKKLIIAMDAAFGMEYLHSK--IVHFDLKCDN
LLVDPQRICKVGDFGLSRIKSG-VRGTLPWMAPELL-SSRVSEKVDVFSFGIALWEILTG
EEP--NIVNNSLRPPVWRILMEQCWSANPDIRPSFTE
>HORVU.MOREX.r3.5HG0443020.1_kinase_478_726
GEQVGQGSCGTVYHALWYGSDVGVKVFSRQE--IQAFRQEVSLMKKLRHPNILLFMGAVT
------IVTEFLPRGSLFRLLQK--MDWRRRVHMALDVARGMNYLHHYPPIIHRDLKSSN
LLVDKNWTVKVADFGLSRLKTTTGKGTPQWMAPEVL-RNPSDEKSDVYSYGVILWELVTQ
KIP--NVGFMNQRLEIWTSLILSCWETDPQSRPSFQE
>HORVU.MOREX.r3.5HG0449270.1_kinase_74_332
GEELGRGEFGVTRRCMDTGEKLACKSISKR-RSIEDVRREVAIMRSLSHVNVVRLRE--F
EDDDSVLVMEVCEGGELFDRIVVRHYTERAAAAVMRTIMEVVQHCHQ-NGVMHRDLKPEN
FLYANA-LLKVIDFGLSVCF-PEIVGSPYYMAPEVLKRN-YGQEIDIWSAGVILYILLCG
VPPFWEIDFEREPPK-AKDLVSMMLENNPYTRLTAQQ
>HORVU.MOREX.r3.5HG0458010.1_kinase_257_499
-EMIASGSCGDLFHGTYFGEDVAVKVLKAEH--WNEFTQEVYILREVHHTNVVRFIGACT
------IITEYMSGGSLYDYVH-V-VDLPTLLKFACDVCRGMCYLHQR--IIHRDLKTAN
LLMDKDHVVKVADFGVARFQMT-ETGTYRWMAPEVI-NHPYDNKADVFSFAIVLWELLTS
KIP--TVRQ-GLRPVLLLDLLQRCWETIPSNRPAFPD
>HORVU.MOREX.r3.5HG0474810.1_kinase_683_881
ANKIGEGGFGSVYKGLLDGTIIAVKQLSSKSQGNREFVNEIGMISALQHPNLVRLYGCCT
EGNQLLLVYEYMENNCLARALFYRALDWPTRRKICLGIARGLAYMHEEIRIVHRDIKASN
ILLDKDLDAKISDFGLAKLNSTKVAGTIGYMAPEYAMRGYLTDKADVYSFGVVALEIVSG
KSNT---------------------------------
>HORVU.MOREX.r3.5HG0477430.1_kinase_93_375
LCKIGSGVSAVVYKAACLGSVVAIKAIDLE-RSLEDVWREAKAMALLSHANVLRAHC--F
TVGSHLVVMPFMAAGSLHSILAGFGLPEPCVAVVLKETLRALCYLHE-QGRIHRDIKAGN
VLVDSDG-VKLADFGVSASILSEMAGTPYWMAPEVIHSHGYGIKADIWSFGITALELAHG
RPPLSPVRMEDAE--IFKDMVSSCLCQEPAKRPSAEK
>HORVU.MOREX.r3.5HG0478260.1_kinase_104_365
GEMIGSGAFGQVYLGMNTGELLAVKQVLIGSKAIRELEEEVKLLKNLSHPNIVRYLG--V
REEGTLILLEFVPGGSIQSLLGKLSFPEAVIRKYTRQILQGLEYLHS-NAIIHRDIKGAN
ILVDNKG-IKLADFGASKQVLATMKGTPHWMAPEVIVGSGHTFSADIWSVGCTVIEMATG
KPPWSEVGTTKSHPPIAKDFLLKCLQKEPELRSSASD
>HORVU.MOREX.r3.5HG0479660.1_kinase_55_276
KQKLGQGGFGPVYRGRLDGREVAVKRLGAGSQGAREFKNEANLLSRVQHRNVVNLLGYCA
HGDEKLLVYEYVPNESLDKILFVRELTWPRRLEVVVGVARGLLYLHEDTPIIHRDIKASN
ILLDERWVAKIADFGMARLFQTRVAGTNGYMAPEYLMHGHLSAKADVFSFGVLVLEILSG
RK-----------------------------------
>HORVU.MOREX.r3.5HG0479880.1_kinase_146_413
HETIGRGTTADIHRATWRGLEVAVKWIRPELNPEAFFAQELDALSRQRHPHVLRLMGACL
------LVTELLSGATLGEWLHRRARPLAQRVSRALEVALAMRHLHEQPRVLHRDLKPSN

VLLDADSRARVADFGHARFLLT-ETGTYVYMAPEVI-RCPYTEKCDVYSFGVMLNELVTG
EHP--DVADGKLRPKLLVDLICRAWDAEPSRRPSFAA
>HORVU.MOREX.r3.5HG0495010.1_kinase_48_326
EQELGRGGYGVVYKGMLNGKMIAVKKLFEMQ--DDQFQKEVTYLIGVKHQNIVQLVGYCA
ESRTRLLCFEYLKNNSLDKHLSESGLEWHIRYDIIRGVCSGLHYLHAE-RIIHLDLKPQN
ILLDDHMMPKLADFGMSRLFITSRGGTFGYMAPEYITNGLITTKSDIFSLGIIIIELMTG
HRRYPDRLEKTLS--VCIVLGLNCLNPDPSKRPSAWD
>HORVU.MOREX.r3.5HG0495030.1_kinase_49_326
DQELGRGGFGVVYKGMLNGKMIAVKKLFEMG--DDQFQNEVTYLIGVKHQNIVQLVGYCA
ESRKRLLCFEYLKNNSLDKHISESGLEWRIRYDIIKGVCSGLHYLHAE-RIVHLDLKPQN
ILLDDSMVPKLADFGMSRLFITSRGGTLGYMAPEYINNGVISTKSDIFSLGTIIIELLTG
SREYPHRLEKTLS--ICIVLGLNCFDLDPHKRPSARD
>HORVU.MOREX.r3.5HG0501470.1_kinase_359_571
SNKLGQGGFGAVYKGTLDGEEIAVKRLSKSSQGVEELKNELALVAKLKHKNLVRLVGVCL
EQQERLLVYEFVPNRSLDQILFKGQLDWGMRHRIIRGIARGLQYLHEDLKVVHRDLKASN
VLLDADMNPKISDFGLARLFTNRVIGTYGYMAPEYLMRGNYSVKSDVFSFGVMVLEIVTG
RKNSD-----TLQSQ---------------------
>HORVU.MOREX.r3.5HG0501480.1_kinase_368_636
SNKLGEGGFGAVYKGTLDGEQIAVKRMSKSSQGLEELRNELALVAKLKHKNLVSLIGVCL
EQQERLLVYEFLPNRSLDLILFKRQLDWAKRHKIIEGIARGLQYLHEDLKVVHRDLKASN
ILLDMNSVPKISDFGLAKIFTNRVVGTHGYMAPEYMMRGNYSVKSDAFSFGVMVLEIVTG
RKNNDSVVDPCMNGSVCVQIGLLCVQENPVDRPTMST
>HORVU.MOREX.r3.5HG0501530.1_kinase_366_630
RNKLGEGGFGAVYKGILDGQEIAVKKLLGTAHGLDQLHNEVLMLAELQHKNLVRLHGFYS
HRDDTLLVYEYIKNGSLDNFLSEGTLVWGQQYNIILGIAKGILYLHEDMRVIHRDLKPNN
ILLDEEMEAKIADFGLARLLTSGAVGTLGYMAPEYAIHGRVSPKIDIFSYGVLVLQIVTR
RRECWAMMDQTLDQHQCLHVGLMCVQAHPDDRPEIS-
>HORVU.MOREX.r3.5HG0501600.1_kinase_319_563
GNKVASGSYGDLFRGTYCSQDVAIKVLKPER--QREFAQEVYIMRKVRHKNVVQFIGACT
------IVTEFMSGGSVYDYLH-I-FKLPALVGVAMDVSKGMSYLHQN--IIHRDLKTAN
LLMDENGMVKVADFGVARVKMT-ETGTYRWMAPEVI-EHPYDHKADVFSFGILLWELLTG
KIP--YVVQKGLRPTILTELLQKCWQQDPAERPDFSE
>HORVU.MOREX.r3.5HG0503580.1_kinase_528_728
TCKIGQGGFGKVYKGLLGGQEVAIKRLSSDSQGTKEFRNEVILIAKLQHRNLVRLLGCCG
EGDEKLLIYEYLPNKSLDATLFRRMLDWTTRFNIIKGVARGLLYLHQDLTIIHRDLKAGN
VLLDVEMKPKIADFGMARIFTQRVVGTYGYMAPEYAMEGVFSTKSDVYSFGVLVLEVVTG
IKRSS--------------------------------
>HORVU.MOREX.r3.5HG0503630.1_kinase_538_732
-CMIGQGGFGKVYKGMVGGQEVAIKRLSRDSQGTKEFRNEVILIAKLQHRNLVRLLGCCS
EGDEKLLIYEYLPNKSLDATLFRKLLDWEIRFSIIKGVARGLMYLHEDFTIIHRDLKVGN
ILLDADLKPKIADFGMARIFTQRVVGTYGYMAPEYAMEGVFSTKSDVYSFGVLLLEVVTG
I------------------------------------
>HORVU.MOREX.r3.5HG0503690.1_kinase_536_730
-CMIGRGGFGKVYKGMLGGQEVAIKRLSRDSQGTKEFRNEVILIAKLQHRNLVRLLGCCS
EGDEKLLIYEYLPNKSLDVTLFRKLLDWATRFSIIKGVSKGLRYLHEDFIIIHRDLKAAN
VLLDADFKPKIADFGMARIFTQHIVGTYGYMAPEYAMEGVFSTKSDVYSFGVLLLEVVTG
I------------------------------------
>HORVU.MOREX.r3.5HG0508800.1_kinase_71_342
RHVIAQGTYGTVYRGTYDGQQVAVKLLDWGETTRTSFKQEVAVWHKLSHPNVTKFVGASM
GTDDN-VVVEYLAGGTLKQYLIRK-LAYKVVVQLALDLSRGLSYLHSR--IVHRDVKTEN

MLLDTQRNLKIADFGVARVEMT-ATGTLGYMAPEVL-DGPYNRKCDVYSFGICLWEIYCC
DMP--DVVHQNLRPDIFANIMRKCWDGNPDKRPDMDE
>HORVU.MOREX.r3.5HG0509880.1_kinase_785_1091
---------SAVYHCKI-GSVAAAKILDTRR--FEYLLGEVRMMNALKHRSIVDIYGHQV
QDDYRIILMEYVKGGSLKGYLTKDHVPVDLAFYIAREVACALLEMHRK--VIHRDIKSEN
VLVDSK-VVKLSDFDRSIPLIAICVGTPCWMAPEVLQEKQYGLEVDIWSYGCFIFEMLT-
-IPYQDIKRKKQRPRILIDLFYQCTRGTASRRPKAEQ
>HORVU.MOREX.r3.5HG0510050.1_kinase_88_356
QNQVASGTFGVVYRGTYDGNDVAVKVLDWGQSSREAFEKEVAVWQKLDHPNVTKFVGASM
GTQKK-VVVEYQHGGTLKTLLFKK-LPYKKVVQLALDMARGLNYLHSQ--IVHRDVKAEN
MLLDRKKSVKIADFGVARVEMT-QTGTLGYMAPEVL-EGPYDHKCDVYSFGVLLWETYCC
ALA--NVVKLGIRPDILSEIMTRCWDGNPDHRPEMAE
>HORVU.MOREX.r3.5HG0510630.1_kinase_804_1012
HNILGSGSFGKVYKGLLSGLVVAIKVLDMQLQA-QSFDIECQVLRVTRHRNLIKILNTCS
THEFRALVLEYMPNGSLETFLHSTHRGFLERLGIMLDVSMAIEYLHHDELILHCDVKPSN
VLFDEEMTAHVADFGIARLLSGSMPGTVGYMAPEYGTLGKASRKSDVFSYGIMLLEVFTG
RRPTDQ------------------------------
>HORVU.MOREX.r3.5HG0511440.1_kinase_283_537
LHELGKGSYGAVYKARDTQELVAVKIISLT-EGYEDIRGEIEMLQQCSHPNVVRYFG--Y
QGEEYLIVMEYCGGGSVADLIGEE-LDEPQIAYICRETLKGLAYLHT-IFKVHRDIKGGN
ILLTEQG-VKLGDFGVAAQL--TFIGTPHWMAPEVIQES-YDGKVDVWALGVSAIEMAEG
MPPRSP-ISSEPAPMLFHDFIAKCLTKDARLRPAAIE
>HORVU.MOREX.r3.5HG0512150.1_kinase_445_693
GEQVGQGSCGTVYHALWYGSDVAVKLFSKQE--VNTFRQEVSLMKKLRHPSIILFMGAVA
------IVTEFLPRGSLFQLLQK--LDPRRKLNMAIDIARGMNYLHNSPTIVHRDLKSSN
LLVDKNWTVKVADFGLSRLKSTNGKGTPQWMAPEVL-RSPSNEKSDVYSYGVVLWELVTQ
KIP--TVGFMDHRLEIWASIIESCWDSDPQRRPSFQE
>HORVU.MOREX.r3.5HG0512370.1_kinase_469_727
LREMGAGAFGTVFHGKWRGTDVAIKRINNSCADITEFWREAAIISKLHHPNILALYGVVN
------TVTEFMVNGSLKKVLSY--LDWRKRLLVAMDAAIGMEYLHSK--IVHFDLKCDN
LLVDPSRICKVADFGLSKMKSG-MRGTLPWMAPELL-SGKVSEKVDVYSFGVVMWEILTG
EDP--GILSDTLRPPVWRKLMEQCWSTEPERRPSFTE
>HORVU.MOREX.r3.5HG0512750.1_kinase_367_625
RKLIGSGTYGCVYEATNTGALCAMKEVNIIPKSLKQLEQEIKFLSQFKHDNIVQYYG--E
TTEDRFIYLEYVHPGSINKYISQHAMTESVVRNFTRHILNGLAFLHS-QKIMHRDIKGAN
LLVDVNG-VKLADFGMAKH-LSSLKGTPYWMAPEVVQATGYDLAVDIWSLGCTIIEMFTG
KPPWSGVLNKD--PPIGKDFLKGCFKRIPSERPTASK
>HORVU.MOREX.r3.5HG0520080.1_kinase_38_327
APCLGKGSFGTVVRARHTGRTVAIKFCDSED--AAELQREAGFLAACGNPYVVGSHGLV-
--DGRLLAMECVAGPSLHAFLR--PLPEPIVCAYMWRLLTGAGMMHR-LGIVHRDLKPSN
ILVAKGGILKICDLGLAMSLRTSDAGTLPYMAPEVLLGKDYDAGADTWSLGCVMAEMLTG
KPLFKDSPDDRTWPEFGYQVLKGLLECNPGKRLTAAA
>HORVU.MOREX.r3.5HG0523860.1_kinase_60_318
GGELGRGEFGVTYLCTETGARMACKSISKR-RTVEDVRREVEIMRHMPHPNIVSLSA--Y
EDEDAVLLMELCEGGELFDRIVARHYTERAAAAVTRTIVEVVQMCHR-NGVIHRDLKPEN
FLYANK-PLKAIDFGLSVFF-PEIVGSPYYMAPEVLKRN-YGPEIDVWSAGVILYILLCG
VPPFWQVDFKRDPPR-AKDLVRRMLDPNPITRLTAAQ
>HORVU.MOREX.r3.5HG0524710.1_kinase_95_351
GSKIGEGAHGKVYKGKYGDQIVAIKVLNSGSKAEDRFIREVNMMCKVKHDNLVKFIGACK
------IVSELLPGMSLKNYLNSQ-LDIHTALGYALNIARAMECLHAN--IIHRDLKPDN

LLLTANRKLKLTDFGLAREEMT-ETGTYRWMAPELYSEKHYTNKVDVYSFGIVLWELLTN
KMP--GAAFKQVRPAFLASIVQSCWVEDPAMRPSFSQ
>HORVU.MOREX.r3.5HG0531390.1_kinase_576_774
-MLIGKGGTSHVYKAQLDGTLYAAKILKPSV--LQEFITEIETVTSLQNENIVSLRGFSF
DN-YFLLVYDYMHQGSLDKALH--SLSWEKRNKIAIHIAKALEFLHHGYSVIHGDVKSAN
ILLSEDFEAQLCDFGLAKKVCTDITGTFGYMAPEYFSHGKVNKKIDVYAFGVVLLEIISG
R------------------------------KP----
>HORVU.MOREX.r3.5HG0535240.1_kinase_803_1073
DSILGSGSFGKVFKGRLNGLVVAIKVLDMQLQA-RSFDVECQVFRMVRHRNLIKILNTCS
NLDFRALVRQYMPNGNLDILLHSICLGFLERLGIMLDVSMAMNYLHHEELILHCDLKPSN
VLFDEEMTAHVADFGIARLLSTSMPGTVGYMAPEYGLLGKASRKSDVYSYGIMILEVFTG
RRPIDQVIDGQLLQGLLFELGLACTTDSPDKRMTMSN
>HORVU.MOREX.r3.5HG0535950.1_kinase_369_637
ENKLGEGGFGTVYKGQLEGLEIAVKRLASHSQGFIEFQNELQLVAKLQHLNLVRLLGCCS
QEEEKILVYEYLPNKSLDYFIFKRLLDWSKLVAIIEGIAHGLLYLHKHLLVIHRDLKPSN
ILLDSEMNPKISDFGLAKIFTRRVIGTYGYMAPEYASKGIFSIKSDVFSFGVIILEILSG
KQNSGQLVDSSFVPHMWINIALLCVQENPADRPTMGD
>HORVU.MOREX.r3.5HG0535960.1_kinase_373_576
ENKLGEGGFGPVYKGHFEGIEIAVKRLDSDSQGFIEFKNEVELIAKLQHRNLVRLMGCCS
QGEEKVLVYEYLPNKSLDFFIFRKLLDWEKRLVIIVGIAEGLLYLHKHLRVIHRDLKPSN
ILLDSTMNAKISDFGLAKIFTRKVVGTYGYMAPEYASHGLFSVKSDVFSFGVLVLEIISG
KKNSH--------------------------------
>HORVU.MOREX.r3.5HG0537810.1_kinase_947_1206
LRELGSGTFGTVYHGKWRGTDVAIKRIKKSCQEAQEFWREAEILSKLHHPNVVAFYGVVK
------TLTEFMVNGSLRHVLQC--PDLRKRLIIAMDAAFGMEYLHSK--IVHFDLKCDN
LLVDHARICKVGDFGLSKIKSG-VRGTLPWMAPELL-SSKVSEKVDVFSFGIVLWEILTG
EEP--NIVNNTLRPPVWRRLMEQCWSPDPSQRPAFTE
>HORVU.MOREX.r3.6HG0549970.1_kinase_302_544
-SKVASGSNGDLYRGSYCIQDVAIKVVRPER--YRDFAQEVYIMRKVRHKNVVQFIGACT
------IITDFMSGGSVYDYLH-S-FKLPEILRVATDISKGMSYLHQN--IIHRDLKTAN
LLMDENKVVKVADFGVARVKMT-ETGTYRWMAPEVI-EHPYDHKADVFSFGIVLWELLTG
KIP--YVVQKGIRPTILGELLQKCWHKDSAERPDFSQ
>HORVU.MOREX.r3.6HG0565330.1_kinase_39_303
DQQIGSGGFAVLYKGIL-GKVIAVKKL-SKT--EKQFHEEVSCLMKAKHKNIVRFLGYCS
DTRQRLLCFEYLPRGSLDKYIASSVLQWKESYQIIKEICEGLHYLHVERRIVHLDLKPTN
ILLDCNMVPKIADFGLSRRFLTEVKGTLGYMAPEFVNNGQITFKSDIYSLGIIITEILTR
QRGYR-RLQESQR--VCTEIAKECSDYNPANRPAIQD
>HORVU.MOREX.r3.6HG0569550.1_kinase_29_293
MEPIGDGATAVVRRARCLGEVVAVKIMNLA-RSVNNASEEVKTMILTDHPNLLSAYC--F
TQDENLIVMPYMAGGSCFHLMKSFGFEEERIAFVLRETLRGLEYLHG-KGHIHRDVKAGN
ILLDQHK-VKLADFGISASVINTLVGTPCWMAPEVMEQKEYDFKADIWSFGITALELANG
HAPFSP-TLQHAPPSLFKRMIGACLIKDPSKRPTARM
>HORVU.MOREX.r3.6HG0570850.1_kinase_148_391
-----QGAFGKLYRGTYIGEDVAIKLLEKPDAQEQQFVQEVMMLSTLRHPNIVRFIGACR
------IITEYAKGGSVRQFLAKS-VPLRLAVKQALDVARGMAYVHAL--FIHRDLKSDN
LLIAADRSIKIADFGVARIEMT-ETGTYRWMAPEMI-QHPYDHKVDVYSFGIVLWELITG
MLP--NVVNKGARPAILTHIMTRCWDANPEVRPPFTE
>HORVU.MOREX.r3.6HG0575140.1_kinase_85_349
----ASGSNSRIYRGIYRQRAVAVKMVRLPERREEQFNSEVSFLSRLRHPNVVQFVAACK
------IITEYMSQGTLRMYLHYS-LSTETVLRLALDVARGMEYLHAQ--VIHRDLKSHN

LLLNDEMRVKVADFGTSCLEGATNMGTYRWMAPEMV-RDPCTRKVDVYSFGIVLWELTTC
LVP--GACEKNARPPLLNNLIKMCWAANPARRPEFSY
>HORVU.MOREX.r3.6HG0579960.1_kinase_604_852
GERIGLGSYGEVYHADWNGTEVAVKKFLDQD--LEQFKCEVRIMSRLRHPNVVLFLGYVT
------ILTEYLPRGSLYRLLHK--VDETRRLKMALDVAKGMNYLHTSPTIVHRDLKSPN
LLVDKNWVVKVSDFGMSRLKSSSTAGTPEWMAPEVL-RNPANEMCDVYSFGVILWELATL
CVP--GVGFQNKRLDIVASIISSCWDNDPSKRPSFSQ
>HORVU.MOREX.r3.6HG0580210.1_kinase_7_165

--PAIVHCDVKSAN
ILLDAEFRAKIADFGLARILISAIGGTFGYMAPEYGYRLKVNEKVDVYSFGVVLLELTTG
RMANDELLDEDIRDPAVFTLGVICTGGQPSVRPSMKD
>HORVU.MOREX.r3.6HG0584740.1_kinase_538_781
GTRVGIGFFGEVFRGIWNGTDVAIKVFLEQD--MEDFCNEISILSRLRHPNVILFLGACM
------LVTEYMEMGSLYYLIHKSKISWRRRLKMLRDICRGLMCMHRL--IVHRDLKSAN
CLVNKHWTVKLCDFGLSRLLSDSSAGTPEWMAPELI-RNPFTEKCDIFSLGVIMWELCTL
SRP--GVANEGARLEILGSLIADCW-TEPEKRPSCQE
>HORVU.MOREX.r3.6HG0590820.1_kinase_32_287
-PRIGEGGHAKVYEGKYKNQNVAIKIVHKGDVVQGRFLREVTMLSRVQHKNLVKFIGACL
------VVTELLVGGSLRKYLVRN-LEPRVAVGFALDIARAMECLHAH--IIHRDLKPEN
LLLTADQTVKLVDLGLAREEMT-ETGTYRWMAPELYSEKHYNHKVDVYSFAIVLWELLHN
RLP--GAAFKNIRPS-LSEILTSCWKEDPSDRPNFTQ
>HORVU.MOREX.r3.6HG0602230.1_kinase_393_648
GKLIGRGTFGHVYAGFNRGQMCAMKEVTLFSKSAKQLDQEISLLSRLQHPNIVRYYG--E
TVEDKLIYLEFVSGGSIHKLLQEYQFGEQAIRNYTKQILLGLAYLHA-TNTVHRDIKGAN
ILVDPKG-VKLADFGMAKH-INSLKGSPYWMAPEVIKNSGCNLAVDIWSLGCTVLEMATS
KPPWSGIGNSKELPPIGKDFIRQCLQRDPSSRPTAVD
>HORVU.MOREX.r3.6HG0603230.1_kinase_28_282
NEVLGRGAVKTVYKAFDEGIEVAWNQVNIDEQCLERLYSEVHLLKSLKHENVMKFYNYWD
DRKKTIVITELFTSGNLRLYRRPR-VDLKAIKNWARQILHGLDYLHSHPPIIHRDLKCDN
IFVNGN-QVKIGDLGLATIM--SVIGTPEFMAPELYDED-YDELVDIYSFGMCMLEMFTL
EYPYNNVSKGVKPAGLVKNFIEKCLVP-ASERLSAKE
>HORVU.MOREX.r3.6HG0608490.1_kinase_13_223
ENKLGEGGFGPVYRGVLGGAEIAVKRLSARSQGAAEFRNEVELIAKLQHRNLVRLLGCCV
EKDEKMLVYEYLPNRSLDAFLFKTQLDWKMRQSIIVGIARGLLYLHEDLKIVHRDLKASN
VLLDNKMNPKISDFGMAMIFTGHVVGTYGYMAPEYAMGGVFSVKSDVFSFGVLVLEILSG
QRNGAE-------------------------------
>HORVU.MOREX.r3.6HG0610010.1_kinase_540_814
TEELGHGRHGSVYKGILDSRVVAVKKLNDVKQGEDEFEAEVSVIGKIYHMNLVRVMGVCS
ERSHRLLVYEYVENGSLAMFLFDKPLLWHQRYKVAAGVAKGLAYLHHEDWIIHCDVKPEK
ILLDMDFDPKISDFGFAKLLMSKVRGTRGYMAPEWVSTAPLTEKLDVYSFGVVLLELVMG
SRVSEDFVDPRLNGDVMLEVAALCLEKERSQRPSMNH
>HORVU.MOREX.r3.6HG0615510.1_kinase_835_1083
GERIGLGSFGEVYRGEWHGTEVAVKKFLQQD--LEEFRAEVRIMKRLRHPNVVLFMGAIT
------IVTEFLPRGSLFRLIHL--LDEKRRLRMALDVARGMNYLHNCPVIVHRDLKSPN
LLVDKNWVVKVCDFGLSRMKSSSTAGTAEWMAPEVL-RNPSDEKCDVFSYGVILWELCTL
QQP--GVGFQSRRLDIVAEIITRCWQTDPRARPSFAE
>HORVU.MOREX.r3.6HG0617920.1_kinase_438_686
GEQVGQGCCGTVYHALWYGSDVAAKVFSKQE--INTFRQEVSLMKKLRHPNIILFMGAVA
------IVTEFLPRGSLFRLLQK--LDPRRRVNMAIDIARGMNYLHSSPTVVHRDLKSPN

```
LLVDKNWTVKVADFGLSRLKSTTGKGTPQWMAPEVL-RSPSNEKSDVFSYGVVLWELATQ
KIP--TVGFMDHRLEIWASMIESCWGSEPQHRPSFQE
>HORVU.MOREX.r3.6HG0618820.1_kinase_272_522
GEHIGSGSFGSVYEAI-DGFFFAVKEVSLLDNAIVQLEHEVSLLSRLEHDNIVQYYG--D
KEDGKLIFLELVTQGSLAALYQKYCLQDSQVSAYTRQILNGLNYLHQ-RNVLHRDIKCAN
ILVDANG-VKLADFGLAKE-MSSSKGTIFWMAPEVAK-APHGPPADIWSLGCTVLEMLTG
KVPYPWIGRGIP-PKIARDFITRCVQSNQNDRPSAAQ
>HORVU.MOREX.r3.6HG0622060.1_kinase_820_1080
IKELGSGTYGSVFHGKWRGCDVAIKRIKASCREIADFWKEAQILSSLHHPNVVSFYGVVR
------TVTEFMVNGSLKQFLRT--IDRRKRVILAMDAAFGMEYLHGK--IVHFDLKCEN
LLVDPQRICKIGDLGLSKVKSG-VRGTLPWMAPELL-KSMVSEKIDVYSFGIVMWELLTG
DDP--DIVNNSLRPQIWKSLMEGSWAGEPAQRPSFTE
>HORVU.MOREX.r3.6HG0623180.1_kinase_28_289
YEEIGQGVSAIVYRALCLAETVAVKVLDFE-RTLNNIMREAQTMILIDHPNVVRALC--F
ANNQTLVVMPYMAGGSCLHIMKVYGFDEAVIATVLREVLRGLEYLHH-HGHIHRDVKAGN
ILVDSRG-IKLGDFGVSACL-DTFVGTPCWMAPEVMEQLGYDFKADIWSFGITALELAHG
HAPFSP-TLQNAPPGLFKQMVAMCLVKEPSKRPTAAK
>HORVU.MOREX.r3.6HG0624460.1_kinase_694_964
-NWIGSGRSGKVYRMCVEGRMVAVKKIWNTQDNEKDFLAEVQILGEIRHTNIVKLLCCIS
SSEAKLLVYEYMENGSLDRWLHGSPLDWPTRLQIAIDSARGLCYMHHDPAIVHCDVKSAN
ILLGPEFRAKIADFGLAQILISVIGGTFGYMAPEYGYRLKVNEKVDIYSFGVVLLELTTG
RVANDELLDEDIRDPAVFTLGVICTVGQPSVRPSMKD
>HORVU.MOREX.r3.6HG0630250.1_kinase_139_389
------------------------------------SARLAVLGHCHHAAIARLYGAAA
SPDGALLAYELVPGAALSSLLRPSLASWHSRLRLAADACDALSYVHLQ-GTVHNRLSSSS
VLVQGALRAKLAHFGSADLARTRIEGTRGYMAPELIAGGSPTRRSDVFALGVLLLELVSG
QEPVRRWVDRRLKDSFLTTLALQCVSKDPGARPDMS-
>HORVU.MOREX.r3.7HG0638110.1_kinase_361_629
ENKLGQGGFGPVYKGMFDGTEVAVKRLAAQSQGLVEFKNEIQLIAKLQHTSLVKLVGCCV
QEEEKMLVYEYMPNRSLDFFIFRGLLDWKKRQHIIEGIAQGLLYLHKHVRIIHRDMKASN
ILLDKDLNPKISDFGMARIFTNRVVGTYGYMAPEYASGGFFSVKSDLFSFGVLLLEIVSG
KRNSSNLVDPTLGHCICVKVALLCVQDNAMDRPTISD
>HORVU.MOREX.r3.7HG0640060.1_kinase_903_1111
DNLLGRGCFGKVFKGVVSGSVVAIKVLDMQLQA-RSFDVECHVLRMARHRNLIRIIINTCS
NLDFRALVLQYMPNGSLEMLLHSTPLGFLERIDILLDVSMAMEYLHFEEVILHCDLKPSN
VLFDQGMIGHVADFGIARLLCASMAGTVGYMAPEYGSFGKASRKSDVFSYGIMLLEVFTR
KRPTDE------------------------------
>HORVU.MOREX.r3.7HG0650300.1_kinase_68_319
IKVIGKGSSGTVQLVRHTGQFFALKVIQLN-IQRKQIAQELKISLSTQCQYVVTCYQ--F
YVNGVIIALEYMDGGSLADFLKRT-VPEAYLAAICKQVLKGLMYLHNEKHVLHRDLKPSN
ILINHRG-VKISDFGVSAIIASTFTGTFNYMAPERISGQKHGYMSDIWSLGLVMLECATG
NFPYPSAVVDQPSPSAFCSFVSACIQKNATDRSSAQT
>HORVU.MOREX.r3.7HG0658560.1_kinase_1011_1271
LRELGSGTFGTVYHGKWRGSDVAIKRISDRCEQKTDFWNEACKLSSLHHPNVVAFYGVVL
------TVTEYMANGSLRQALQI--FDRRRRLLIVMDVAFGMEYLHGK--IVHFDLKSDN
LLVDPQRICKVGDLGLSKVKSG-VRGTLPWMAPELL-SSLVSEKVDVFSFGIVMWELLTG
EEP--DIVNNTLRPLVWRSLMEQCWSAEPTERPSFTE
>HORVU.MOREX.r3.7HG0659090.1_kinase_817_1088
KYVIGTGAHGTVYKATLSGEVYAIKKLAISASSYKSMIRELKTLGKVRHRNLIKLKEFWV
RGDSGFILYDFMEHGSLYDVLHGTSLDWSMRYNIALGTAHGLAYLHHDPAIIHRDIKPSN
```

ILLNKDMVPRISDFGIAKIMTTGVVGTTGYMAPELAFSTRNSIKTDVYSYGVVLLELITG
KTAVDSVCDPALLD-VVLRLALRCTANEPSRRPSMVD
>HORVU.MOREX.r3.7HG0659780.1_kinase_518_719
-CMIGLGGFGKVYKGTLGGQEVAIKRLSMDSQGVNEFKNEVILISKLQHKNLVRLLGCCE
KGDEKLLIYEYLPNKSLDATLFRKLLDWGTRLTIIKGVAKGLLYLHEDLTIIHRDLKAGN
VLLDAEMKPKIADFGMARIFTQRVVGTFGYMAPEYAMQGIISTKSDIYSFGVLLLEIVTG
MKRSS---------------------------------
>HORVU.MOREX.r3.7HG0666850.1_kinase_791_1038
GERVGLGSFGEVYRGEWHGTEVAVKKFLQQD--LEELKAEVRIMKRLRHPNVVLFMGAVT
------ILTEFLPRGSLFRLIRQ--LDERKRIRMALDVARGMNYLHNCPVVVHRDLKSPN
LLVDKNWVVKVCDFGLSRIKSSSTAGTAEWMAPEVL-RNPSDEKCDVFSYGVILWELCTL
LQP--GVGFQERRLDIIAEIIERCWQTDPKTRPSFSE
>HORVU.MOREX.r3.7HG0669620.1_kinase_813_1015
KYIIGTGGQGTVYKATLSGEVYAVKKLVGHAKIHGSMIREMNTLGQIRHRNLVKLKDVLF
KREYGLILYEFMDNGSLYDVLHAANLEWRIRYDIALGTAHGLAYLHNDPAIIHRDIKPKN
ILLDKDMVPHISDFGIAKLITTGIVGTVGYMAPEMAFSTRSTIEFDVYSYGVVLLELITR
KMALD---------------------------------
>HORVU.MOREX.r3.7HG0673840.1_kinase_281_540
ENFIGKGSLTEVYKGVLDDKAVAVKRFVKIKEDDMAFRREAEVIRVAVHNNILRLTGYCM
ERKERLLVYPFMENLSLSSNLENQTLDWAKRMKIALGVAHALEYLHDNPPIIHRDIKAAN
VLLNGNFEAVLGDFGLAMIMTTEIQGTVGYMAPEYRSTGKASTKTDVYGYGVLLLEIVTG
KGPDFEIVDPNLDRALLMNISLLCTQEEAELRPTMSR
>HORVU.MOREX.r3.7HG0675780.1_kinase_103_369
ANKLGEGGFGPVYRGVMCGAEVAVKRLSVRSQGAAEFRNEVELIAKLQHRNLVRLLGWCA
ERDEKLLVYEYLPNGSLDAFLFKSELDWNTRHNIIIGIARGLLYLHEDLKIIHRDLKPSN
VLLDNKMNPKISDFGLARIFTGRVVGTYGYMAPEFVMDSLFSVKSDVFGFGVLLLEILSG
QRNGIEFIDRVLGQSACFQVGLLCVQDDPDIRPTMSN
>HORVU.MOREX.r3.7HG0675830.1_kinase_99_366
ANKLGEGGFGPVYRGVLGGSEIAVKRLSARSQGAAEFRNEVELIAKLQHRNLVRLLGWCA
ERDEKLLVYEYLPNRSLDAFLFKSQLDWKTRHGIILGIARGLLYLHEDLKVVHRDLKASN
VLLDNKMRPKISDFGMAKIFTGRVVGTYGYMAPEFVMEGVFSVKSDVFSFGVLLIEILGG
KRNGAEFMDPALGRAACFHVGLLCVQDDPDLRPTMSS
>HORVU.MOREX.r3.7HG0676820.1_kinase_17_278
YEEVGEGVSATVYRALCLNTFVAIKVLDLE-KCMDGIRREVQTMSLINHPNLLRACC--F
ANDHHLVVMPFMAAGSALHIIKNFGFEEAVIATLLREVLKALVYLHS-QGHIHRDVKAGN
ILIDTNG-VKLGDFGVSACM-NTFVGTPCWMAPEVMQQLGYDYKADIWSFGITALELAHG
HAPFSP-TLQNAPPGLFKDLVSTCLVKDPQKRPSSEK
>HORVU.MOREX.r3.7HG0696770.1_kinase_143_387
-----QGAFGKLYRGTYNGMDVAIKLLERPEAQEQQFVQEVRMLAELRHPNIVKFVGACR
------IVTGYAKGGSVRNFLNRS-VPLKLAVKQALDVARGMAYVHGL--FIHRDLKSDN
LLISGDKSIKIADFGVARIEMT-ETGTYRWMAPEMI-QHPYNQKVDVYSFGIVLWELITG
TLP--NVVNKGVRPAILGEIMTRCWDANPDVRPPFTD
>HORVU.MOREX.r3.7HG0707460.1_kinase_265_509
GEKITSGSSADLYRGTYNGLDVCIKILRSVH--EVEFLQQALMLRRVKHENILTFYGTCT
------TITEYMPGGDLYGFIH-V-LDLFLILRIAISISKGMEYLHQH--IIHRDLKTAN
ILMGDNHVVKIADFGVARLGMT-ETGTYRWMAPEII-NHPYDHKADVFSFAIILWELITL
KVP--NVRQ-GLRLEILSKLTEQCWDEDPDIRPVFTE
>HORVU.MOREX.r3.7HG0718040.1_kinase_691_976
ENVIGSGGSGKVYRIHLEGRMVAVKKIWNGRDADKEFESEVKVLGNIRHNNIVKLLCCIS
SQDVKLLVYEYMENGSLDRWLHGAPLDWPTRLAIAIDSAKGLSYMHHDQSIVHRDVKSSN

ILLDPEFHAKIADFGLARMLVSAIGGTFGYMAPEYASRLRVNEKVDVYSFGVVLLELVTG
KVANDDVVDEHIRDPIVFTLAVICTGENPPARPTMKE
>HORVU.MOREX.r3.7HG0722010.1_kinase_106_348
-----AGANSRIYRGIYKQRAVAVKMVRIPERAEDQFNSEVAFLSRLYHPNIVQFIAACK
------IITEYMSQGTLRMYLNYS-LSPETILKLALDISRGMEYLHAQ--VMHRDLKSQN
LLLNDEMRVKVADFGTSCLETK-NKGTYRWMAPEMI-KEPYTRKVDVYSFGIVLWELTTC
LLP--GAAEKNLRPPLLNNLIKRCWSANPARRPEFSY
>HORVU.MOREX.r3.7HG0738770.1_kinase_224_485
DNLIGKGGHAEVYKGQLDGQFVAVKRLTKGG--ISDFLSELGIIAHVNHPNAAQLLGFSV
EG----LVLQFSPHGSLASLLHAKALRWKARFNIALGVAEGLFYLHEGRHIIHRDIKASN
ILLTEDYQPQISDFGLAKWLCTPIEGTFGYMAPEYFMHGIINEKTDVFAYGVLLLELVTG
RKAVD-LVDPSLD--MTLAVASMCIHHSANLRPSMKS
>HORVU.MOREX.r3.7HG0739050.1_kinase_152_396
-----QGAFGKLYRGTYNGEDVAIKLLEKPEAQEQQFVQEVMMLSTLRHPNIVRFIGACR
------IITEYAKGGSVRQFLAKS-VPLRLAVKQALDVARGMAYVHAL--FIHRDLKSDN
LLISADKSIKIADFGVARIEMT-ETGTYRWMAPEMI-QHPYDHKVDVYSFGIVLWELMTG
MLP--NVVNKNARPAILSHIMTRCWDANPEVRPSFNE
>HORVU.MOREX.r3.7HG0744210.1_kinase_832_1090
LQELGSGTFGTVYHGKWRGSDVAIKRINDRCQERNDFWNEASNLADLHHPNVVAFYGVVL
------TVTEYMVNGSLRTALLS--LDRRKRLIIAMDTAFGMEYLHNK--IVHFDLKSDN
LLVDPQRICKVGDLGLSKVKSG-VRGTLPWMAPELL-SSLVSEKVDVFSFGIVLWELLTG
EEP--DIVSNTLRPQVWRSLMEQCWATEPSERPSFTQ
>HORVU.MOREX.r3.7HG0745900.1_kinase_51_311
KHRIGRGPFGDVWIATHDDHEVAVKMVRDDQ--FDE------VFGKCGLGNVCFLHGIS-
TQNGRLIAMKFYE-GSIGDRMA--RLPLSDVLRYGADLARGVLDLHS-RGIFVLNLKPCN
FLLDDNDHAVLGDFGIPSLLLPQRLGTPNYMAPEQWQRGPISYETDSWGFACSILEMFSG
VQPWGDVVLKKEKPIFVENVLFGCFEYDFRDRPLMSD
>HORVU.MOREX.r3.7HG0748850.1_kinase_502_771
REEVGRGGSGIVYRGLLDKRVIAVKNLIDITKHEEEFRAEMSVIGRINHMNLVRTWGFCS
EAKRKLLVYEFVENESLDRYLFADLLAWSERFRIALGSARALAYLHHEEWVLHCDVKPEN
ILLTRDFEAKIADFGLAKLYFSHMRGTDGYMAPEWAMNLPINAKVDVYSYGVVLLELVAG
RRISSDIVDAKLHGQAMITTAVSCLAEERSRRPSMNE
