# Supplementary material for: Dormancy Versus Germination: 3D Protein Modeling and Evolutionary Analyses Define the Roles of Genetic Variants in the Barley MKK3 Enzyme
Source: Int J Mol Sci. 2026 Jan 5;27(1):530. doi: 10.3390/ijms27010530 (PMC12787138; doi:10.3390/ijms27010530)
Supplement: Supplementary file 1 [file ijms-27-00530-s001.zip › ijms-4033614-Dormancy Versus Germination_Supplementary Table S1.pdf]

Supplementary Table S1. Purifying selection across Triticeae MKK3 orthologues (site-by-site_FEL_analysis_0.1).

Partition	codon	alpha	beta	alpha=beta	LRT	p-value	Total branch length	class
1	4	1.101	0.000	0.421	3.278	0.0702	0.698	Purifying
1	7	17.975	0.000	1.417	11.480	0.0007	2.348	Purifying
1	11	2.086	0.000	0.576	4.600	0.0320	0.955	Purifying
1	12	3.161	0.000	0.344	5.462	0.0194	0.571	Purifying
1	27	5.525	0.639	1.640	3.991	0.0457	2.718	Purifying
1	33	27.255	0.000	0.900	14.892	0.0001	1.492	Purifying
1	38	1.466	0.000	0.233	3.491	0.0617	0.386	Purifying
1	42	7.394	0.000	1.199	10.498	0.0012	1.986	Purifying
1	47	5.966	0.000	0.741	10.493	0.0012	1.228	Purifying
1	49	5.823	0.000	0.259	5.617	0.0178	0.429	Purifying
1	53	0.955	0.000	0.202	3.001	0.0832	0.335	Purifying
1	54	2.265	0.000	0.568	5.665	0.0173	0.942	Purifying
1	57	1.178	0.000	0.240	3.080	0.0793	0.398	Purifying
1	58	5.123	0.000	0.332	4.521	0.0335	0.549	Purifying
1	61	3.359	0.271	0.636	2.932	0.0868	1.055	Purifying
1	65	5.878	0.000	0.518	7.765	0.0053	0.859	Purifying
1	66	44.082	0.000	0.213	5.088	0.0241	0.354	Purifying
1	67	2.854	0.000	0.269	4.159	0.0414	0.446	Purifying
1	69	9.746	0.286	0.837	4.483	0.0342	1.387	Purifying
1	71	6.878	0.285	1.134	7.296	0.0069	1.879	Purifying
1	78	5.853	0.649	1.546	4.387	0.0362	2.562	Purifying
1	80	1.930	0.000	0.233	3.395	0.0654	0.386	Purifying
1	88	2.789	0.000	0.246	4.511	0.0337	0.407	Purifying
1	90	7.578	0.000	0.216	5.673	0.0172	0.358	Purifying
1	91	2.282	0.000	0.275	4.309	0.0379	0.455	Purifying
1	92	5.071	0.000	0.263	4.947	0.0261	0.436	Purifying
1	96	5.225	0.000	0.457	7.939	0.0048	0.757	Purifying
1	97	40.341	0.667	1.731	7.441	0.0064	2.868	Purifying
1	98	1.594	0.000	0.209	3.341	0.0676	0.347	Purifying
1	101	9.900	0.000	0.411	9.335	0.0022	0.680	Purifying
1	102	5.338	0.000	0.793	7.916	0.0049	1.315	Purifying
1	103	1.886	0.000	0.481	4.757	0.0292	0.797	Purifying
1	107	2.680	0.000	0.250	3.816	0.0508	0.415	Purifying
1	108	3.483	0.356	0.878	2.757	0.0969	1.454	Purifying
1	110	4.849	0.000	0.370	8.048	0.0046	0.612	Purifying
1	116	1.806	0.000	0.412	5.683	0.0171	0.682	Purifying
1	119	1.175	0.000	0.243	3.053	0.0806	0.402	Purifying
1	120	1.905	0.000	0.257	3.775	0.0520	0.426	Purifying
1	122	2.282	0.000	0.275	4.309	0.0379	0.455	Purifying
1	126	1.178	0.000	0.240	3.080	0.0793	0.398	Purifying

1	127	2.886	0.000	0.251	3.971	0.0463	0.416	Purifying
1	128	4.705	0.000	0.682	9.878	0.0017	1.130	Purifying
1	130	1.333	0.000	0.254	3.150	0.0759	0.421	Purifying
1	132	1.522	0.000	0.252	3.681	0.0550	0.417	Purifying
1	133	1.466	0.000	0.238	3.445	0.0635	0.394	Purifying
1	137	1.985	0.000	0.410	5.630	0.0177	0.679	Purifying
1	139	9.376	0.328	1.015	4.812	0.0283	1.682	Purifying
1	143	1.081	0.000	0.202	3.246	0.0716	0.335	Purifying
1	144	3.387	0.000	0.489	5.215	0.0224	0.810	Purifying
1	145	3.046	0.000	0.426	8.180	0.0042	0.705	Purifying
1	148	1.353	0.000	0.244	2.816	0.0934	0.404	Purifying
1	151	1.175	0.000	0.242	3.045	0.0810	0.400	Purifying
1	152	1.289	0.000	0.227	3.541	0.0599	0.376	Purifying
1	154	5.759	0.000	0.261	4.588	0.0322	0.432	Purifying
1	156	10000	0.257	0.513	2.718	0.0992	0.849	Purifying
1	157	2.794	0.000	0.401	7.543	0.0060	0.665	Purifying
1	160	1.057	0.000	0.209	2.825	0.0928	0.346	Purifying
1	167	1.333	0.000	0.253	3.185	0.0743	0.419	Purifying
1	172	1.242	0.000	0.369	4.598	0.0320	0.612	Purifying
1	173	3.654	0.000	0.784	8.492	0.0036	1.299	Purifying
1	176	7.124	0.000	0.548	9.578	0.0020	0.909	Purifying
1	178	1.905	0.000	0.257	3.775	0.0520	0.426	Purifying
1	186	1.178	0.000	0.240	3.080	0.0793	0.398	Purifying
1	189	1.286	0.000	0.279	3.923	0.0476	0.462	Purifying
1	191	5.745	0.000	0.213	4.982	0.0256	0.352	Purifying
1	196	2.048	0.000	0.502	5.011	0.0252	0.831	Purifying
1	197	1.792	0.000	0.620	3.743	0.0530	1.028	Purifying
1	198	1.038	0.000	0.232	2.951	0.0858	0.385	Purifying
1	205	3.904	0.000	0.259	3.995	0.0456	0.429	Purifying
1	207	0.955	0.000	0.202	3.001	0.0832	0.335	Purifying
1	211	1.455	0.000	0.203	3.746	0.0529	0.336	Purifying
1	217	1.149	0.000	0.196	3.072	0.0797	0.325	Purifying
1	223	1.450	0.000	0.427	4.602	0.0319	0.707	Purifying
1	225	4.491	0.000	0.449	7.248	0.0071	0.744	Purifying
1	228	3.308	0.000	0.382	5.623	0.0177	0.632	Purifying
1	231	1.558	0.000	0.197	3.430	0.0640	0.326	Purifying
1	233	1.031	0.000	0.199	2.983	0.0842	0.330	Purifying
1	237	1.289	0.000	0.227	3.541	0.0599	0.376	Purifying
1	238	1.624	0.000	0.527	4.464	0.0346	0.874	Purifying
1	241	4.463	0.358	0.968	3.642	0.0563	1.604	Purifying
1	248	1.455	0.000	0.246	3.373	0.0663	0.407	Purifying
1	251	3.678	0.289	0.772	3.679	0.0551	1.280	Purifying
1	252	1.722	0.000	0.496	4.784	0.0287	0.822	Purifying

1	253	1.031	0.000	0.196	2.900	0.0886	0.325	Purifying
1	256	1.209	0.000	0.286	2.938	0.0865	0.474	Purifying
1	257	2.540	0.000	0.358	6.453	0.0111	0.594	Purifying
1	260	2.219	0.000	0.249	3.527	0.0604	0.412	Purifying
1	261	4.372	0.000	0.582	9.418	0.0021	0.965	Purifying
1	262	1.466	0.000	0.238	3.445	0.0635	0.394	Purifying
1	263	1.178	0.000	0.240	3.080	0.0793	0.398	Purifying
1	264	1.081	0.000	0.214	3.144	0.0762	0.354	Purifying
1	266	3.036	0.000	0.550	6.819	0.0090	0.912	Purifying
1	267	5.923	0.000	0.341	5.106	0.0238	0.565	Purifying
1	268	1.198	0.000	0.218	3.505	0.0612	0.360	Purifying
1	271	2.646	0.000	0.495	5.864	0.0155	0.821	Purifying
1	273	1.476	0.000	0.194	3.833	0.0502	0.321	Purifying
1	277	0.986	0.000	0.199	2.931	0.0869	0.329	Purifying
1	280	3.863	0.000	0.235	4.085	0.0433	0.389	Purifying
1	282	1.289	0.000	0.219	3.612	0.0574	0.363	Purifying
1	284	1.289	0.000	0.227	3.541	0.0599	0.376	Purifying
1	288	5.923	0.000	0.341	5.106	0.0238	0.565	Purifying
1	291	7.412	0.270	0.792	4.511	0.0337	1.313	Purifying
1	302	3.059	0.000	0.749	7.993	0.0047	1.241	Purifying
1	304	3.387	0.000	0.344	6.056	0.0139	0.570	Purifying
1	306	5.086	0.000	0.874	7.877	0.0050	1.448	Purifying
1	310	0.754	0.000	0.169	2.898	0.0887	0.280	Purifying
1	317	8.716	0.000	0.562	9.180	0.0024	0.932	Purifying
1	318	3.387	0.000	0.364	5.895	0.0152	0.602	Purifying
1	319	1.464	0.000	0.219	3.591	0.0581	0.363	Purifying
1	320	1.468	0.000	0.417	4.537	0.0332	0.691	Purifying
1	321	1.656	0.000	0.206	3.329	0.0681	0.342	Purifying
1	333	0.875	0.000	0.190	2.973	0.0847	0.315	Purifying
1	334	3.874	0.000	0.424	8.541	0.0035	0.703	Purifying
1	336	6.862	0.000	0.499	9.790	0.0018	0.828	Purifying
1	342	1.076	0.000	0.185	3.418	0.0645	0.307	Purifying
1	344	1.558	0.000	0.207	3.329	0.0681	0.342	Purifying
1	346	2.890	0.000	0.236	3.655	0.0559	0.391	Purifying
1	348	1.467	0.000	0.306	2.950	0.0859	0.506	Purifying
1	349	3.151	0.000	0.346	5.499	0.0190	0.574	Purifying
1	354	1.289	0.000	0.195	3.300	0.0693	0.322	Purifying
1	358	2.741	0.000	0.302	3.281	0.0701	0.500	Purifying
1	367	18.566	0.000	0.628	10.637	0.0011	1.041	Purifying
1	368	1.642	0.000	0.210	3.449	0.0633	0.348	Purifying
1	373	5.786	0.000	0.420	7.525	0.0061	0.696	Purifying
1	378	1.294	0.000	0.273	3.188	0.0742	0.452	Purifying
1	380	2.741	0.000	0.302	3.281	0.0701	0.500	Purifying

1	400	285.538	0.335	0.975	9.901	0.0017	1.615	Purifying
1	402	2.631	0.000	0.611	5.821	0.0158	1.013	Purifying
1	406	2.733	0.000	0.223	3.861	0.0494	0.370	Purifying
1	413	1.886	0.000	0.408	5.410	0.0200	0.676	Purifying
1	418	1.455	0.000	0.246	3.373	0.0663	0.407	Purifying
1	421	1.770	0.000	0.618	3.724	0.0536	1.024	Purifying
1	422	2.325	0.000	0.576	5.341	0.0208	0.954	Purifying
1	425	2.361	0.000	0.429	6.249	0.0124	0.711	Purifying
1	428	2.035	0.000	0.604	3.805	0.0511	1.001	Purifying
1	431	7.514	0.288	0.824	4.383	0.0363	1.366	Purifying
1	434	3.925	0.316	0.992	3.586	0.0583	1.645	Purifying
1	436	1.333	0.000	0.255	3.157	0.0756	0.423	Purifying
1	440	1.890	0.000	0.490	4.863	0.0274	0.813	Purifying
1	442	3.151	0.000	0.346	5.499	0.0190	0.574	Purifying
1	445	28.898	0.276	0.770	4.711	0.0300	1.276	Purifying
1	446	43.854	0.000	0.739	14.793	0.0001	1.225	Purifying
1	447	1.905	0.000	0.358	6.027	0.0141	0.594	Purifying
1	448	3.053	0.000	0.423	6.625	0.0101	0.701	Purifying
1	451	1.333	0.000	0.253	3.185	0.0743	0.419	Purifying
1	454	3.624	0.282	0.613	3.109	0.0779	1.016	Purifying
1	456	9.306	0.335	0.886	4.812	0.0283	1.469	Purifying
1	457	1.133	0.000	0.205	3.428	0.0641	0.340	Purifying
1	459	1.198	0.000	0.245	3.258	0.0711	0.406	Purifying
1	460	0.943	0.000	0.207	2.728	0.0986	0.343	Purifying
1	462	18.064	0.295	0.770	5.805	0.0160	1.276	Purifying
1	463	1.445	0.000	0.243	3.308	0.0689	0.403	Purifying
1	467	4.146	0.337	0.899	3.038	0.0813	1.490	Purifying
1	468	2.983	0.000	0.297	5.422	0.0199	0.492	Purifying
1	469	2.408	0.000	0.741	6.251	0.0124	1.227	Purifying
1	482	1.297	0.000	0.167	3.596	0.0579	0.276	Purifying
1	492	1.076	0.000	0.178	3.499	0.0614	0.295	Purifying
1	496	2.773	0.324	0.867	3.157	0.0756	1.437	Purifying
1	500	3.181	0.000	0.525	6.445	0.0111	0.869	Purifying
1	502	18.110	0.000	0.597	12.114	0.0005	0.989	Purifying
1	503	0.000	1.315	0.789	2.940	0.0864	1.307	Diversifying
1	511	7.799	0.000	0.894	12.019	0.0005	1.481	Purifying
1	515	2.947	0.000	0.241	4.350	0.0370	0.399	Purifying
1	519	5.956	0.299	0.667	3.802	0.0512	1.106	Purifying
