# Supplementary material for: Dormancy Versus Germination: 3D Protein Modeling and Evolutionary Analyses Define the Roles of Genetic Variants in the Barley MKK3 Enzyme
Source: Int J Mol Sci. 2026 Jan 5;27(1):530. doi: 10.3390/ijms27010530 (PMC12787138; doi:10.3390/ijms27010530)
Supplement: Supplementary file 1 [file ijms-27-00530-s001.zip › Supplementary Materials_FigsS1-S5_MH-070126_MAPK_corrected.pdf]

Supplementary Materials

Dormancy Versus Germination: 3D Protein Modeling and Evolutionary Analyses Define the Roles of Genetic Variants in the Barley MKK3 Enzyme

Maria Hrmova [1,*], Christoph Dockter [2], Flavia Krsticevic[2], Morten Egevang Jørgensen[2], Birgitte Skadhauge [2] and Geoffrey B. Fincher [1]

[1] School of Agriculture, Food and Wine, Waite Research Institute, The University of Adelaide, Waite Campus, Glen Osmond, SA 5064, Australia; geoffrey.fincher@adelaide.edu.au
[2] Carlsberg Research Laboratory, J. C. Jacobsens Gade 4, 1799 Copenhagen V, Denmark.; christoph.dockter@carlsberg.com (C.D.); birgitte.skadhauge@carlsberg.com (B.S.); flavia.krsticevic@carlsberg.com; morten.jorgensen@carlsberg.com
* Correspondence: maria.hrmova@adelaide.edu.au

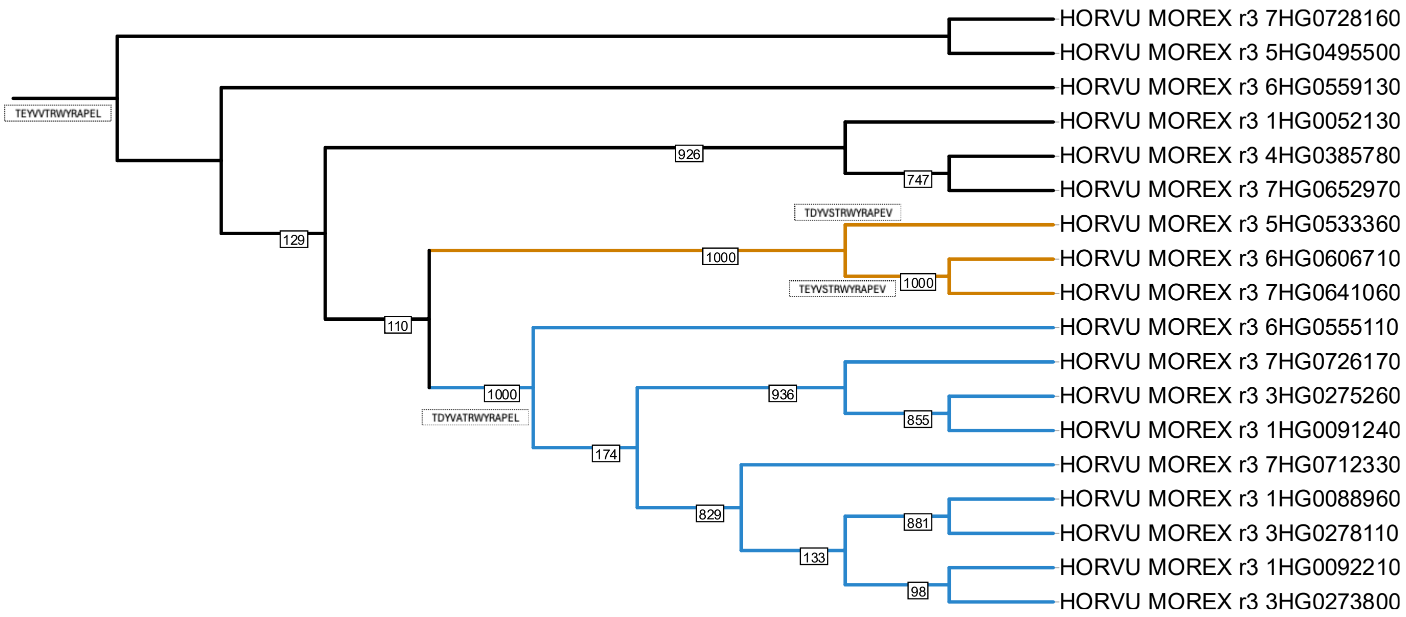

Supplementary Figure S1. Phylogenetic tree of MAPKs identified in MorexV3 using the conserved MAPK activation-loop motif.

Blue branches correspond to MAPKs with the strict TDYVATRWYRAPEL motif, orange branches represent MAPKs carrying XDYVSTRWYRAPEV variants, and black branches denote TEY-type MAPKs with the TEYVVTRWYRAPEL motif. Together, these three motif classes capture the full activation-loop diversity of barley MAPKs and recover the expected TEY–TDY structure of plant MAPK families.

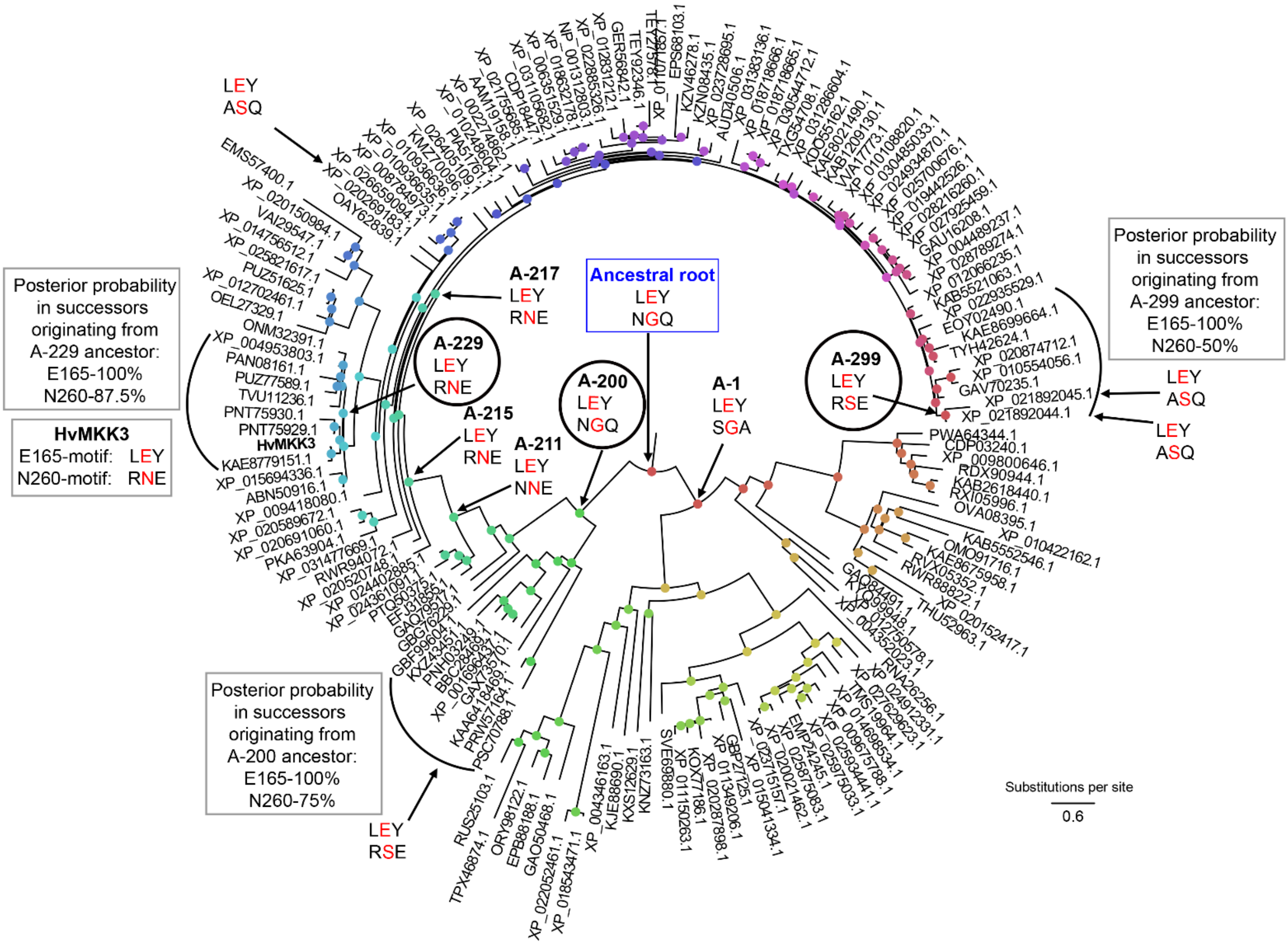

Supplementary Figure S2. Rooted tree of representative MKK3 enzymes (150 in total), annotated by NCBI accessions, with HvMKK3 indicated in bold (accession A0A140JZ13).

Ancestral sequence reconstruction identified 149 ancestors, with ancestral nodes color-coded, using the HvMKK3 sequence as the search input. Residues corresponding to E165 (LEY motif in HvMKK3) and N260 (RNE motif in HvMKK3) or equivalent residues in ancestral nodes are indicated. The ancestral root carries LEY and RSE motifs. The equivalent residues corresponding to E165 and N260, highlighted in red, are indicated for selected ancestral nodes. In three instances, we provide posterior probability values for successors in clusters originating from Ancestor 229 (where HvMKK3 positions), and Ancestors 200 and 299. The coloring of node circles reflects the order starting from ancestral root 151 to ancestor 299, shown across the fire/brick-yellow-yellow/green-green-green-cyan-blue-purple hues. Substitutions per site scale are indicated.

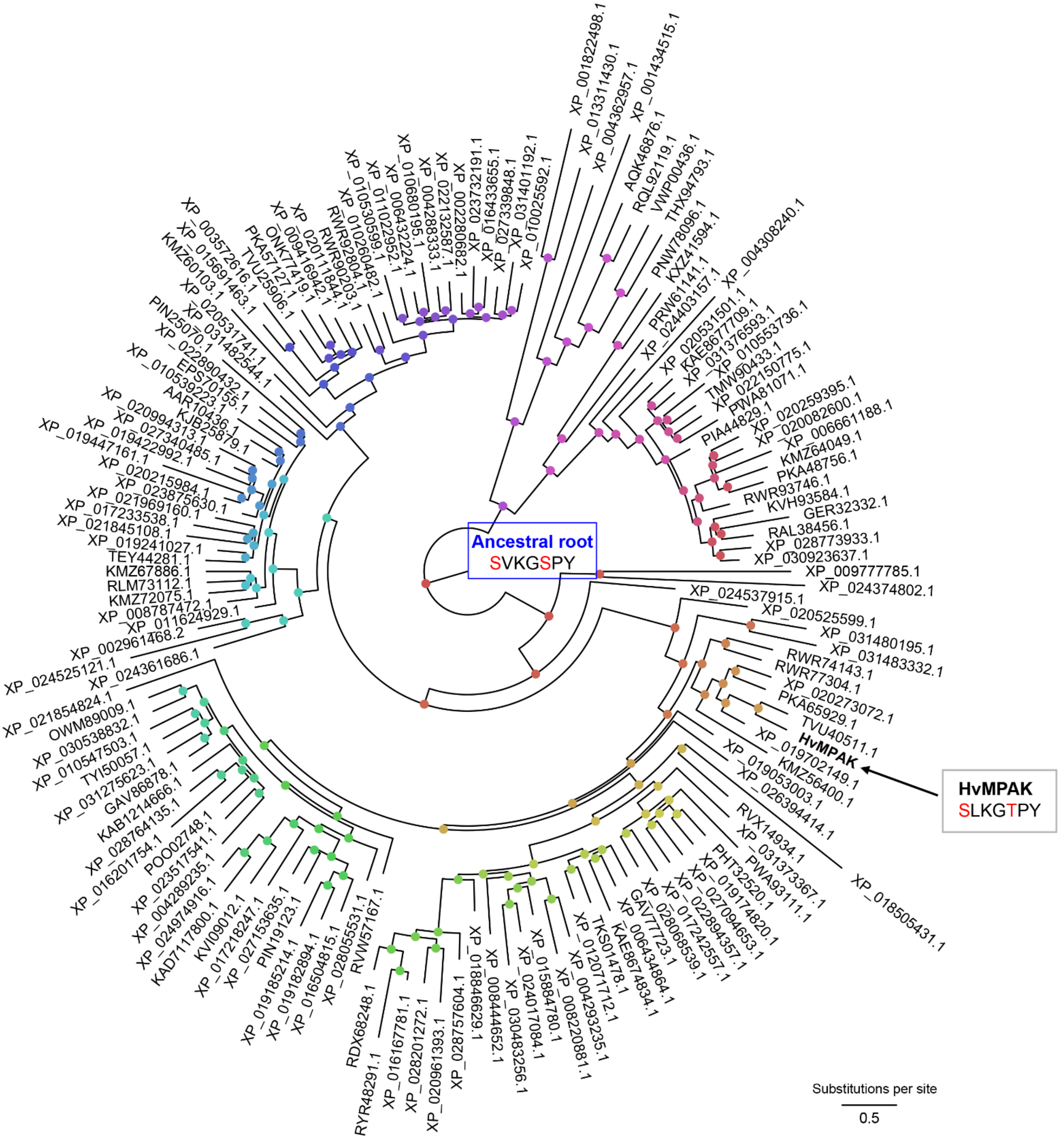

Supplementary Figure S3. Rooted tree of representative MAPK enzymes (150 in total) annotated by NCBI accessions with HvMAPK indicated in bold (accession A0A8I6YLP6), containing the SLKGTPY motif with phosphorylatable residues highlighted in red.

Ancestral sequence reconstruction identified 149 ancestors, with ancestral nodes color-coded, using the HvMAPK sequence as the search input. Reconstruction shows that the SVKGSPY phosphorylation site is present in the Ancestral root and has evolved through several predecessors, with the SLKGTPY, SLKGSPY, SFKGTPY, SVKGSPY, SLKGTPH, SLKGSPY, SMKGTPY, and SIKGSPY phosphorylation motifs. The coloring of node circles is described in the legend to Supplementary Figure S3. Substitutions per site scale are indicated.

```
>HvMKK3; 3D model in AlphaFold Structure Database (accession A0A140JZ13); UniProt accession A0A140JZ13
MAGLEELKKKLQPLLFDDSDKGGVSTRVPFPEDTCDSYVVSDGGTINLLSRSFGEYNINEHGFHKRSTGPEEPDTSEKVYRCASEDMHIFGPIGNG
ASSVVQRAIFIPVHRILALKKINIFEKEKRQQILNEMRTLCEASCYPGLVEFQGAFYMPDSGQISIALEYMDGGSLADVIKVKKSIPEPVLAHMLL
KVLLGLKYLHEARHLVHRDLKPANILVNLKGEAKITDFGVSAGLDNTMAMCATFVGTVTYMSPERIRNENYSYAADIWSLGLTILECATGKFPYNV
NEGPANLMLQILDDPSPAPPEDAYTPEFCSFINDCLRKDADARPTCEQLLSHPFIKRYEETGVDLAAYVRGVVNPTERLKQIAEMLAVHYYLLFNG
SEGPWNHMKMFYREESSFSFSGNVYVGQSAIFDTLSNIRKKLKGDRPREKIVHVVEKLHCRANGETEIAIRVSGSFITGNQFLIFGEGLQAEGMPS
LDEIDIDIPSKRVGQFREQFTVHPGTSMGCYYIAKQDLYIIQS

>HvMAPK; UnProt accession A0A8I6YLP6
MPWWKRSAHRPSSASASTPASPARASTSRIPRHDGAGDLPRLTRQRRLRHVDDIEVGASALRLDDPHAAPPSSSSYPARRDAVWSGLATASSTPIS
RSPSNMMEAAPARSSSTPMLLPHPLPLPHQDDSPCRGPGRPLPSPRMFDGDCNGSADFLGVADTGGDRPSTFPRFMPQTVQKIHEHNDLRSAGTHG
ATCGQRRKTYKEKFQDSPSDTLNFRLNIPAKSAPSSGFSSPVQSPRRLSNVDLSSAAISIQGSNILSAPSPWSSDQSGSSPPSTSPEKFVGGQERS
PRSSPLRSPALRSRYPSAPPSPMRSNLFPENHTSRSEGNPNPNLHPLPLPPVSMSPKQTNFSHPSVPKVETPSMAGQWQKRKLIGSGTYGCVYEAT
NRHTGALCAMKEVNIIPDDAKSVESLKQLEQEIKFLSQFKHDNIVQYYGSETTEDRFYIYLEYVHPGSINKYISQHCGAMTESVVRNFTRHILNGL
AFLHSQKIMHRDIKGANLLVDVNGVVKLADFGMAKHLSTAAPNLSLKGTPYWMAPEVVQATLVKDVGYDLAVDIWSLGCTIIEMFTGKPPWSGLEG
PAAMFKVLNKDPPIPDNLSSEGKDFLKGCFKRIPSERPTASKLLEHPFIQNSNHCTQHVSVHSPAGNKSPDAGHCSREKKSWKTESCVRGKQTNTN
GETSSSRCSGSLGHRPKAPTSLDTHSLSPPPMSYKSSSGSAAHNTPNSMHFSIGYPQPSPLPKPNGKESLNMFSY

>HvMAPK; 3D model in Swiss-Model Depository (accession A0A8I6YLP6); UniProt accession A0A8I6YLP6
VETPSMAGQWQKRKLIGSGTYGCVYEATNRHTGALCAMKEVNIIPDDAKSVESLKQLEQEIKFLSQFKHDNIVQYYGSETTEDRFYIYLEYVHPGS
INKYISQHCGAMTESVVRNFTRHILNGLAFLHSQKIMHRDIKGANLLVDVNGVVKLADFGMAKHLSTAAPNLSLKGTPYWMAPEVVQATLVKDVGY
DLAVDIWSLGCTIIEMFTGKPPWSGLEGPAAMFKVLNKDPPIPDNLSSEGKDFLKGCFKRIPSERPTASKLLEHPFIQNSNH
```

Supplementary Figure S4. Protein sequences of HvMKK3 and HvMAPK, including their AlphaFold Structure Database/UniProt and the Swiss-Model Depository accessions.

Catalytic residues in HvMKK3 and HvMAPK are in bold red and blue letters, phosphorylation motifs are underlined, the candidate phosphorylasable T and S residues are highlighted cyan, and E165Q and N260T mutations are highlighted in magenta. DFG activation segments preceding phosphorylation motifs are in italics.

A RMSF plot of backbone Cα atoms in HvMKK3/HvMPAK model complexes

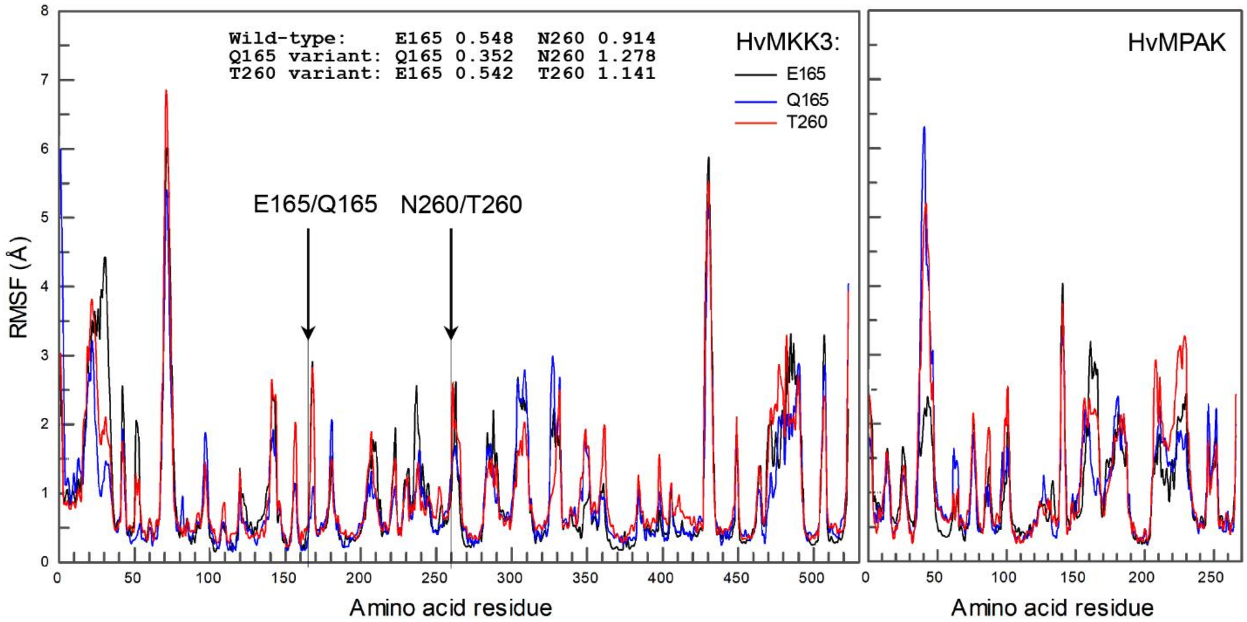

B Sequence of wild type HvMKK3/HvMPAK complex and secondary structures

```
MAGLEELK KKLQPLLF DDSDKGGV STRVPFPE DTCDSYVV SDGGTINL LSRSFGEY NINEHGFH KRSTGPEE PDTSEKVY RCASEDMH IFGPIGNG ASSVVQRA  104
IFIPVHRI LALKKINI FEKEKRQQ ILNEMRTL CEASCYPG LVEFQGAF YMPDSGQI SIALEYMD GGSLADVI KVKKSIPE PVLAHMLL KVLLGLKY LHEARHLV  208
HRDLKPAN ILVNLKGE AKITDFGV SAGLDNTM AMCATFVG TVTYMSPE RIRNENYS YAADIWSL GLTILECA TGKFPYNV NEGPANLM LQILDDPS PAPPEDAY  312
TPEFCSFI NDCLRKDA DARPTCEQ LLSHPFIK RYEETGVD LAAYVRGV VNPTERLK QIAEMLAV HYYLLFNG SEGPWNHM KMFYREES SFSFSGNV YVGQSAIF  416
DTLSNIRK KLKGDRPR EKIVHVVE KLHCRANG ETEIAIRV SGSFITGN QFLIFGEG LQAEGMPS LDEIDIDI PSKRVGQF REQFTVHP GTSMGCYY IAKQDLYI  520
IQS                                                                                                                  523

AGQWQKRK LIGSGTYG CVYEATNR HTGALCAM KEVNIIPD DAKSVESL KQLEQEIK FLSQFKHD NIVQYYGS ETTEDRFY IYLEYVHP GSINKYIS QHCGAMTE  104
SVVRNFTR HILNGLAF LHSQKIMH RDIKGANL LVDVNGVV KLADFGMA KHLSTAAP NLSLKGTP YWMAPEVV QATLVKDV GYDLAVDI WSLGCTII EMFTGKPP  208
WSGLEGPA AMFKVLNK DPPIPDNL SSEGKDFL KGCFKRIP SERPTASK LLEHPFIQ NS                                                    266

HHHHHHHH HHTCCCCC CCCCCCCC CCCCCCCC EEEEETTT EEEEEETT TEEEEEET TEEEEECC CCCCCCCC EEEECCCH HHEEEEEE EEECCCCE EEEEEEHH  104
HTEEEEEE EEETTCHH HHHHHHHH HHHHTTTT TCTTECCE EEEEEETT TTEEEEEE ECCTTEEH HHHHHHHC CCCHHHHH HHHHHHHH HHHHHHHT CCCCCCCC  208
CHHHEEEC TTCCEEEC CTTTHHHC CCCHHHHT TCHHHHHC CCHHHHTT CCCCTHHH HHHHHHHH HHHHHCCC CCCCTTCH HHHHHHHH HCCCCCCC TTTCCHHH  312
HHHHHHHT CCCCCCCC CHHHHHTC HHHHHHHC CCCCHHHH HHTTCCHH HHHHHHHH HHHHHHHH HHHCCHHH HHHHHHHC CTTCEEEE TTEEEECH HHHHHHHH  416
HHHHHHHC CCTTTCEE EEEEEEEE EEEETTEE EEEEEEEE EECCCCEE CCCCCCCT TCCCHHHC CTTCCCCC CEEEEEEE EEEECCCT TEEEEEEE EEEEEECC  520
CCE                                                                                                                  523

EEEEEEEE EETTEEEE EEEETTTC CEEEEEEE CCCTTCET TEETTHHH HHHHHHHH HCCETTEC CEEEEECC CCCEEEEE CCCTTCEH HHHHHTTC CCCCHHHH  104
HHHHHHHH HHHHHHHH TTCCCCCC TTCEEECT TCCEEECC TTTTTCCC CCCCCCCC CCCHHHCC CCCCCCCC CCCCCCCH HHHHHHHH HHHHHHHH CCCTTTTC  208
CHHHHHHH HHHTCCCC CCCCCHHH HHHHHHHT CCCHHHCC CHHHHHHC HHHHCCCC CC                                                    266
```

C All-Cα atom motions of HvMKK3/HvMPAK model complexes (5,000 models in ten clusters)

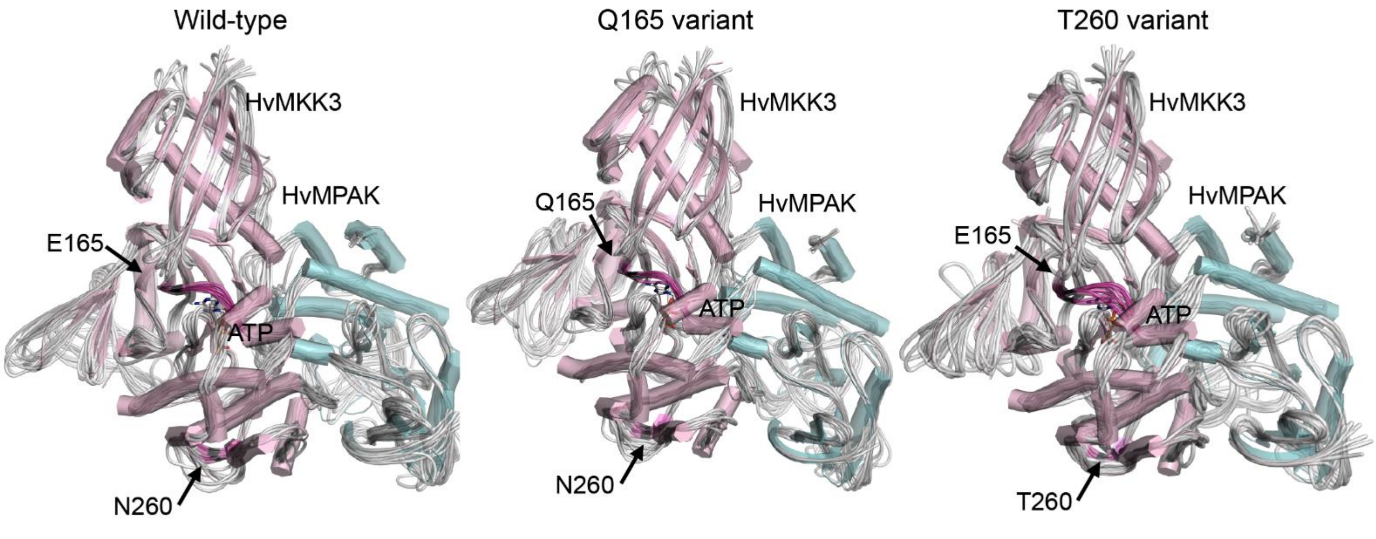

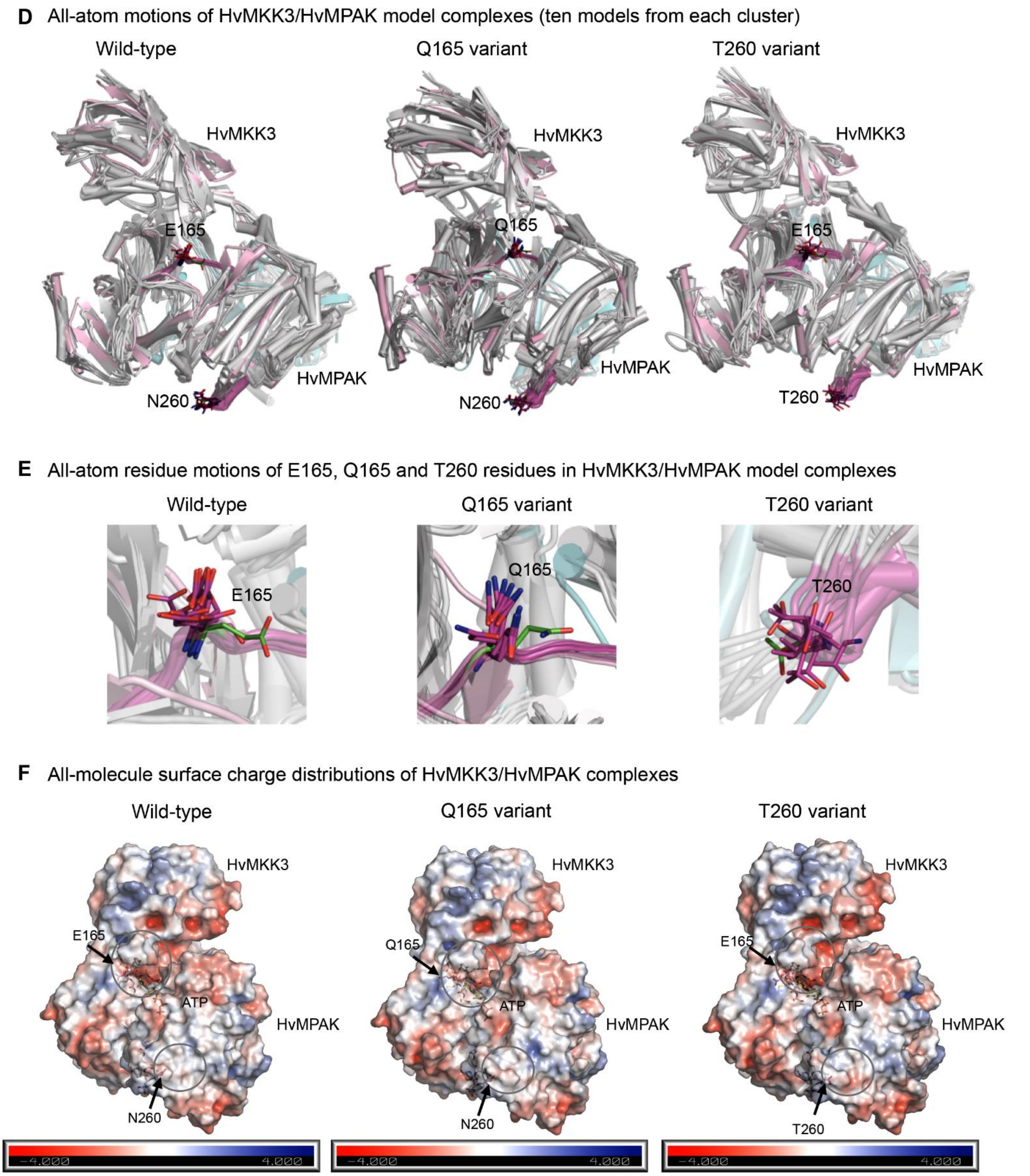

Supplementary Figure S5. Root mean square fluctuation (RMSF) profiles of HvMKK3/HvMAPK model complexes.

(**A**) RMSF plot of backbone Cα atoms in wild-type with E165 and N260 (black trace), and Q165 (blue trace) and T260 (red trace) variant model complexes. Positions of residues explored in this work, E165/Q165 and N260/T260, are marked by arrows. Averaged values for 165 and 260 residues in each complex are indicated. Thin black lines point to positions of E165/Q165 and N260/T260 in the

RMSF plots. For clarity, plots of HvMKK3 or HvMAPK molecules are shown in separate panels, although both molecules were subjected to molecular dynamics simulations as a single entity.

(**B**) Protein sequence of the HvMKK3 (regular types)/HvMAPK (underlined) model complex and disposition secondary structure, predicted by the NetSurfP-3.0 method [1]. The positions of E165 and N260 residues, and associated secondary structures, are highlighted in bold red and magenta, respectively. Secondary structures of HvMKK3 and HvMAPK are indicated in regular and underlined characters, respectively, with the following specification: H, 4-turn α-helix; C, random coil; E, extended ß-sheet; T, H-bonded 3-, 4-, 5-turn.

(**C**) Cartoon representations of all-Cα atom motions of 5,000 model complexes (subdivided in ten clusters: cluster 1-1482 model complexes, cluster 2-973, cluster 3-831, cluster 4-561, cluster 5-302, cluster 6-422, cluster 7-160, cluster 8-101, cluster 9-113, cluster 10-55) superposed on HvMKK3 (pink)/HvMAPK (cyan) starting complexes. RMSD values are between 1.83–2.05 Å, 1.83–2.05 Å, and 1.83–2.05 Å for the wild-type, Q165, and T260 complexes, respectively. Positions of ATP molecules (cpk sticks) are shown. Secondary structures of complexes (magenta) and those carrying the E165/Q165 and N260/T260 residues (black dots) are shown.

(**D**) Cartoon representations of all-atom motions of ten superposed HvMKK3 (pink)/HvMAPK (cyan) model complexes; RMSD values are listed in panel C. Secondary structures (highlighted in magenta and in panel B), carrying E165/Q165, and N260/T260 residues (in magenta sticks), are indicated. The E165, Q165, and T260 residues of the starting structures are in cpk green sticks.

(**E**) Detailed views of all-atom motions of the E165, Q165 and T260 residues of ten representative superposed model complexes of HvMKK3 (pink)/HvMAPK (cyan), shown in panel D. The E165, Q165, and T260 residues of starting structures are in cpk green sticks.

(**F**) All-molecule surface charge distributions of HvMKK3/HvMAPK complexes calculated by Adaptive Poisson-Boltzmann Solver (pdb2pqr method, grid spacing 50, Connolly surface), which solves the equations of continuum electrostatics. Views of surface morphologies colored by electrostatic potentials in wild-type (left), Q165 (centre) and T260 (right) variant complexes are based on those calculated by HDOCK. Positions of ATP molecules (yellow cpk sticks) are shown. Surface morphologies of complexes are colored by electrostatic potentials, and follow scales shown on underlying bars (white, neutral; blue, +4 kTe^{-1}; red, −4 kTe^{-1}). The positions E165, Q165 and T260 residues and surrounding secondary structures (shown in panel B) are circled.

Reference

1. Høie, M.H.; Kiehl, E.N.; Petersen, B.; Nielsen, M.; Winther, O.; Nielsen, H.; Hallgren, J.; Marcatili, P. NetSurfP-3.0: accurate and fast prediction of protein structural features by protein language models and deep learning. *Nucleic Acids Res.* **2022**, *50*, W510–W515.
